# A Genome-Wide CRISPR Screen Identifies Factors Regulating Pluripotency Exit in Mouse Embryonic Stem Cells

**DOI:** 10.3390/cells11152289

**Published:** 2022-07-25

**Authors:** Chen Gao, Xiaolan Qi, Xin Gao, Jin Li, Yumin Qin, Yunjun Yin, Fei Gao, Tao Feng, Sen Wu, Xuguang Du

**Affiliations:** 1 State Key Laboratory of Agrobiotechnology, College of Biological Sciences, China Agricultural University, Beijing 100193, China; chengao@cau.edu.cn (C.G.); 18810380180@163.com (X.G.); 15703277337@163.com (J.L.); yumin_qin0507@163.com (Y.Q.); kayla_yyj@163.com (Y.Y.); gaofei2020019@cau.edu.cn (F.G.); 2 State Key Laboratory of Veterinary Etiological Biology, College of Veterinary Medicine, Lanzhou University, Lanzhou Veterinary Research Institute, Chinese Academy of Agricultural Sciences, Lanzhou 730046, China; xiaolan.qi@cau.edu.cn; 3 Sanya Institute of China Agricultural University, Sanya 572000, China; fengtao@cau.edu.cn

**Keywords:** mouse embryonic stem cells, CRISPR/Cas9, *Nanog* reporter, genome-wide screen, pluripotency

## Abstract

Pluripotency maintenance and exit in embryonic stem cells is a focal topic in stem cell biology. However, the effects of screening under very stringent culture conditions (e.g., differentiation medium, no leukemia inhibitory factor, no chemical inhibitors such as PD0325901 and CHIR99021, and no feeder cells) and of prolonging culture for key factors that regulate pluripotency exit, have not yet been reported. Here, we used a genome-wide CRISPR library to perform such a screen in mouse embryonic stem cells. Naïve NANOG-GFP mESCs were first transfected with a mouse genome-wide CRISPR knockout library to obtain a mutant mESCs library, followed by screening for two months in a strict N2B27 differentiation medium. The clones that survived our stringent screening were analyzed to identify the inserted sgRNAs. In addition to identifying the enriched genes that were reported in previous studies (*Socs3*, *Tsc1*, *Trp53*, *Nf2*, *Tcf7l1*, *Csnk1a1*, and *Dhx30*), we found 17 unreported genes, among which *Zfp771* and *Olfr769* appeared to be involved in pluripotency exit. Furthermore, *Zfp771* knockout ESCs showed a differentiation delay in embryonic chimera experiments, indicating *Zfp771* played an important role in pluripotency exit. Our results show that stringent screening with the CRISPR library can reveal key regulators of pluripotency exit.

## 1. Introduction

Embryonic stem cells (ESCs) are multipotent cells that are produced by the inner cell mass in the blastocyst stage. The molecular mechanisms underlying pluripotency can be studied using ESCs [1]. Mouse embryonic stem cells (mESCs) have remarkable potential for self-renewal in vitro and differentiation into all cell types in the adult organism [2,3]. The maintenance and molecular mechanisms of mESCs regulatory networks have been well established in previous studies [1,4,5]. Several vital signaling pathways, such as JAK/STAT3 [6], WNT [7], and FGF-MAPK [8], maintain self-renewal of mESCs in vitro. Several studies have reported on mESCs pluripotency exit. Leeb et al. (2014) performed a genome-wide screen for self-renewing exit, revealing the involvement of zinc finger protein *Zfp706* and the RNA-binding protein, Pum1, in the differentiation pathway [9]. Li et al. (2018) reported that the loss of two mTORC1-negative regulators, Tsc1/2 and Gator1, can generate opposing phenotypes through differential regulation of Gsk3 activity [10]. Recent developments in CRISPR library technology have provided the means to further interrogate genetic factors that are involved in mESCs exit, and the ability of CRISPR/Cas9 technology to edit genes rapidly and accurately has led to its increasing use in gene-targeting research [11,12,13,14,15,16,17]. In recent years, researchers have created genome-wide sgRNA libraries to perform large-scale screens for specific phenotypes, achieving CRISPR/Cas9-mediated complete gene knockouts, in contrast to the short-term knockdown that is possible with RNAi screens [18,19]. Additionally, CRISPR/Cas9 has the advantage of better consistency across different cell lines.

In this study, we isolated NANOG-GFP mESCs from the NANOG-GFP knock-in mouse and used NANOG-GFP expression as a convenient pluripotency reporter to facilitate our screening. Building on the work of previous large-scale studies employing RNAi and PB transposon-based techniques to screen mESCs differentiation pathways [9,10,20,21,22], we optimized the combination of CRISPR library and PB transposon technology to generate NANOG-GFP CRISPR mutant cells representing the entire mouse genome, revealing novel genes affecting pluripotency exit.

## 2. Materials and Methods

### 2.1. Cell Culture

mESCs were cultured on feeder cells or gelatin in the 2i (CHIR99021, PD0325901)/LIF medium and were differentiated in the N2B27 medium. 2i/LIF medium: 500 mL of Neurobasal medium (Gibco, Shanghai, China, 21103049), 500 mL of DMEM/F12 medium (Gibco, 11330057) that was supplemented with 10 mL N2 (Thermo Fisher Scientific, 17502048), 20 mL B27 (Thermo Fisher Scientific, 12587010), 1 μM PD0325901 (Selleck, S1036), 3 μM CHIR99021 (Selleck, S1263), 0.1 mM β-mercaptoethanol (Gibco, 21986), 1% penicillin/streptomycin (Gibco, 15104122), and 10^6^ unit/L LIF (Millipore, ESG1106). N2B27 medium: 500 mL of Neurobasal medium (Gibco, 21103049), 500 mL of DMEM/F12 medium (Gibco, 11330057) supplemented with 10 mL N2 (Thermo Fisher Scientific, 17502048), 20 mL B27 (Thermo Fisher Scientific, Shanghai, China, 12587010), 0.1 mM β-mercaptoethanol (Gibco, 21986), and 1% penicillin/streptomycin (Gibco, 15104122).

The feeder cells were cultured in DMEM (Gibco, 11960) that was supplemented with 10% fetal bovine serum (Gibco, 10099), 1 mM sodium pyruvate (Gibco, 11360070), 1% nonessential amino acids (Gibco, 11140050), and 1% penicillin/streptomycin (Gibco, 15104122). All the cell lines were incubated at 37 °C in a humidified atmosphere containing 5% CO_2_ and were negative for mycoplasma contamination.

### 2.2. NANOG-GFP Reporter mESCs

NANOG-GFP mice (B6-Nanogtm1Hoch/J, Stock No: 016233/NANOG-GFP) were purchased from the Jackson Laboratory. Available online: https://www.jax.org/strain/016233 (accessed on 23 July 2022). The NANOG-GFP reporter for mESCs, in which one copy of the coding region of the *Nanog* was replaced by a GFP-IRES-Puro-pA element via homologous recombination. The 3.5 days mouse blastocysts were flushed out of the uterus with PBS (containing 1% bovine serum albumin). They were inoculated in a 96-well plate with feeder cells that were seeded one day in advance and cultured in 2i/LIF medium to derive NANOG-GFP mESCs. The expression of GFP and the acquisition of puromycin resistance provided a realistic response to the pluripotency status of mESCs.

### 2.3. Establishment of Single-Gene Knockouts in NANOG-GFP mESCs

Single-gene knockout NANOG-GFP mESCs were obtained by gene editing with pCRISPR-sg6-gene, pCRISPR-S10, and pCAG-PBase plasmids [23]. After electroporation, the cell-containing medium was diluted 10–100 times and seeded onto 10 cm culture dishes that were coated with feeders one day before the experiment. The clones were selected with geneticin (350 μg/mL) for 5 days and then were collected for genotype identification.

### 2.4. Library Construction and Screening

NANOG-GFP mESCs were electroporated with pCRISPR-S10 and pCAG-PBase to obtain Cas9-expressing clones (NANOG-GFP-Cas9 mESCs), which were expanded for sgRNA library transfection. NANOG-GFP-Cas9 mESCs (5 × 10^8^ cells) were electroporated with a PB CRISPR sgRNA library [23] that was derived from GeCKOv2 [16] to obtain a mutant cell pool. About 50% of the cells survived electroporation, resulting in 2.5 × 10^8^ cells, of which about 3 × 10^7^ independent positive cells were drug-resistant. Considering that the PB plasmid library contains 130,000 sgRNAs covering the entire genome of mESCs [24], we obtained a >200-fold coverage mutant cell pool, which was expanded further for use in screening. The mutant cells pool was seeded onto 10-cm plates in N2B27 medium without LIF, 2i, or feeder cells for 8 weeks. The positive cells were selected via puromycin or FACS. Genomic DNA was extracted from the expanded positive cells and subjected to PCR amplification of the sgRNA fragment for deep sequencing analysis.

### 2.5. Quantitative RT-RCR Analysis

The knockout (KO) cell lines were seeded onto 6-well plates at 2.5 × 10^5^ cells/well and incubated for 2 days in N2B27 medium. The cells with the 2i/LIF medium were used as the control. The Majorbio RNA kit (Majorbio Bio-Pharm Technology Co., Ltd., Shanghai, China) was used to extract the total RNA. For each sample, 1 μg of total RNA was transcribed into complementary DNA (cDNA) using the Trans Script First-Strand cDNA Synthesis SuperMix (TransGen Biotech, Beijing, China). Quantitative PCR (qPCR) was conducted using 2×RealStar Green Power Mixture (Genestar, A311-10) on the LightCycler 480 (Roche, Basel, Switzerland). The primers that were used for qPCR are listed in Table S1. Expression levels were normalized to GAPDH.

### 2.6. RNA-seq

RNA-seq was performed on the knockout cell lines that were cultured in the 2i/LIF medium or in the N2B27 differentiation medium for 5 days. mRNA was enriched by A-T complementary pairing with the ploy-A tail of the mRNA using magnetic beads with Oligo (dT). The mRNA was subsequently broken into short fragments by adding fragmentation buffer, and the first strand was synthesized using six-base random hexamers as a template. The purified double-stranded cDNA was then end-repaired, A-tailed, and ligated with sequencing connectors, followed by fragment size selection using AMPure XP beads. The final cDNA library was enriched via PCR. After construction, the library was initially quantified using Qubit 2.0, diluted to 1 ng/μL, and then the insert size of the library was measured using an Agilent 2100 bioanalyzer. After optimizing the insert size, qPCR was performed using a Bio-Rad CFX 96 fluorescence PCR instrument to accurately quantify the effective concentration of the library (>1 ng/μL). Clusters were generated on cBot using the TruSeq PE Cluster Kit v3-cBot-HS (Illumina) reagent. The double-end sequencing program (PE150) was then run on the Illumina sequencing platform to obtain 150 bp double-end sequencing reads. We preprocessed the raw data using Trimmomatic for quality filtering [25]. After removing joint sequences that were contained in the reads, the data were filtered by length, removing reads with lengths less than 50 nt, or with only one end.

### 2.7. GO Enrichment and KEGG Analysis

GO annotations were downloaded from Ensembl’s Biomart database. Available online: http://asia.ensembl.org (accessed on 23 July 2022). The GO enrichment analysis of differentially expressed genes (DEGs) was performed using topGO software 2.36.0 [26]. KEGG (Kyoto Encyclopedia of Genes and Genomes) is the main public database for pathway investigation. Available online https://www.kegg.jp (accessed on 23 July 2022) [27], and was used to analyze the above DEGs with cluster Profiler software 3.12.0. by Yu et al. (Guangzhou, China).

### 2.8. Flow Cytometry

Flow cytometry was performed using FACS CAliburTM flow cytometer (BD, San Jose, CA, USA) to detect the changes in GFP expression after the cells were cultured for different periods under differentiating conditions. The cells were washed with PBS, digested into single cells with TryPLE (Gibco, 12605010), and resuspended in the medium for FACS. The data were analyzed using the Flowjo software v10, using the non-fluorescent mESCs as gating controls for the flow cytometry data.

### 2.9. 8-Cell Embryo Chimerism Experiment

A total of 20 cells of NANOG-GFP mESCs or Zfp771-KO mESCs were injected into 8-cell mouse embryos (ICR mice) and cultured in vitro to blastocyst. For the in vitro chimerism assay, the embryos were observed using a microscope to detect the presence of fluorescent cells.

## 3. Results

### 3.1. CRISPR Library Screening of NANOG-GFP mESCs in a Differentiation Medium

To facilitate screening for pluripotency exit genes, we isolated NANOG-GFP mESCs from the NANOG-GFP knock-in mouse (Figure S1A–C). *Nanog* is the core factor that maintains the naïve state of mESCs [28,29,30]. Both GFP and the puromycin resistance gene of NANOG-GFP are under the control of the *Nanog* promoter [31,32,33,34,35].

**Figure 1:**
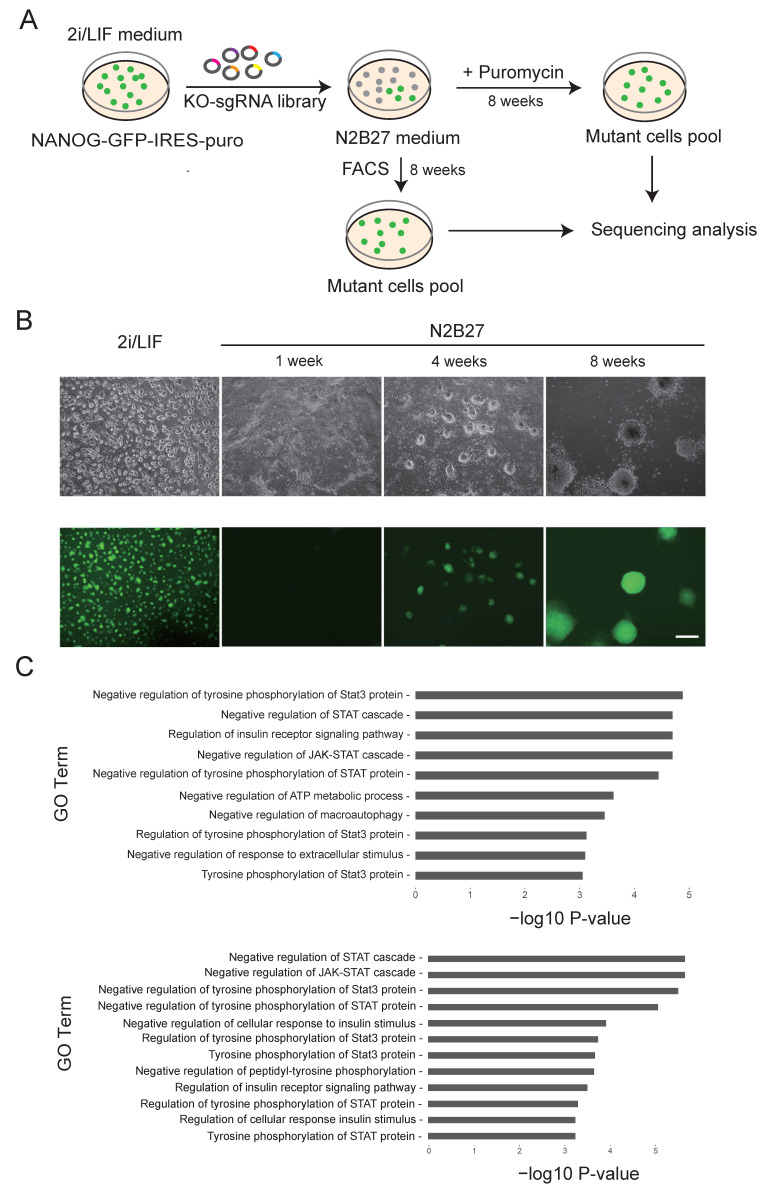
Using the CRISPR library to screen NANOG-GFP mESCs clones in the N2B27 differentiation medium. (**A**) The experimental flow chart shows the strategy of screening with puromycin or FACS sorting. (**B**) Cell morphology of NANOG-GFP mESCs at different screening time points in differentiation medium. (**C**) GO analysis pathways of candidate genes that were obtained in sequence analysis.

To produce a mutant mESCs library with genome-wide knockouts, we first transfected NANOG-GFP mESCs with a Cas9 expression gene cassette, resulting in cloned NANOG-GFP-Cas9 mESCs. We transfected 5 × 10^8^ NANOG-GFP-Cas9 mESCs with a PB CRISPR sgRNA library (Figure S1A) [23]. After drug selection for 5 days, we obtained a mutant cell pool that contained 200-fold coverage of the original sgRNA library. Since mESCs readily exit from pluripotency and undergo differentiation in N2B27 medium without feeder cells, 2i, or LIF [9,10,22], we used the above NANOG-GFP mutant mESCs in five parallel screening experiments. The cells were screened in a differentiation medium until stable clonal cells could be readily cultured. After 8 weeks of screening, we collected puromycin-resistant and GFP-positive NANOG-GFP-Cas9 mESCs via FACS, resulting in 2–5 × 10^4^ positive clones for each experimental group, which were expanded for further experiments (Figure 1A,B). In the control group, GFP expression in NANOG-GFP-Cas9 mESCs was substantially downregulated after two days. The NANOG-GFP-Cas9 mESCs did not survive for five days in the differentiation medium. The results of the control group demonstrated the reliability of this experimental design, consistent with previous descriptions (Figure S1D) [9,10,22]. In the work of Li et al. (2018) and Leeb et al. (2014), the differentiation medium screening time was 48 h and 7–10 days, respectively [9,10]. In comparison, as a strict screening condition, our 8 weeks of screening in the differentiation medium increased the possibility of finding authentic positive clones that could grow in the differentiation medium.

For the five experimental groups, we also isolated individual clones for analysis. We found that each clone contained 7–10 PB-sgRNAs (Figure S2). Immunofluorescence staining and qPCR showed variable increases in pluripotency gene expression in these clones (Figure S3A). Karyotyping analysis revealed that these clones all had a normal karyotype (Figure S3B). The fact that these positive survival clones were pluripotent indicates that our stringent screening strategy is feasible.

To find genes that affect the pluripotency exit of mESCs, we next used 10^8^ expanded positive cells from each of the five experimental groups for genomic DNA extraction, PCR, and deep sequencing to detect sgRNA (Figure S3C). Gene Ontology (GO) analysis showed that the sgRNAs were concentrated in biological processes such as negative regulation of the JAK-STAT signaling pathway, the regulation of STAT3 protein tyrosine phosphorylation, and the regulation of the insulin receptor signaling pathway (Figure 1C). Fittingly, these biological processes are critical for the regulation of pluripotency exit [6,36], supporting the objectives of our screen.

### 3.2. Candidate Genes in the Screen and Their Function in Exit from Pluripotency

**Figure 2:**
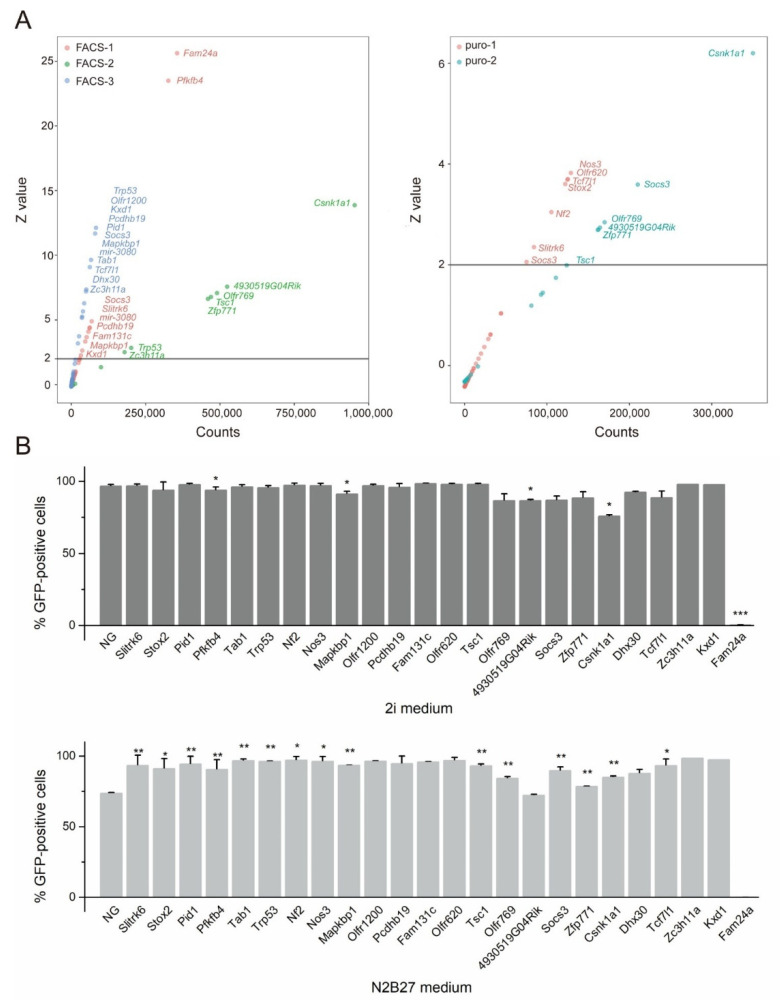
Candidate genes were obtained by screening cells. (**A**) Validated genes that were obtained from five screening experiments. The vertical coordinate Z-value represents the standard score of a set of data. Scatters above 2 represent significant genes, and the numbers in the horizontal coordinate represent the reads that were obtained from high-throughput sequencing. (**B**) GFP expression in single knockout cells after 24 h differentiation in 2i/LIF medium or N2B27 differentiation medium. Data are shown as the mean ± SEM (*n* = 3 independent experiments). Statistical analysis was performed using the Student’s *t*-test. * *p* < 0.05, ** *p* < 0.01. NG: NANOG-GFP mESCs.

To generate a list of candidate genes for pluripotency exit, we performed a pooled overlap of the genes that were measured (Figure S3C). We selected 24 candidate genes for single gene knockout validation based on (1) the frequency of recurrence in parallel experimental groups, (2) Z-score values that were greater than 2 for statistical analysis, and (3) the ranking order of the genes from the screen (Figure 2A, Table S2). The 24 candidate genes are as follows: *Fam131c*, *Fam24a*, *Kxd1*, *Mapkbp1*, *Nf2*, *Nos3*, *Olfr1200*, *Olfr620*, *Olfr769*, *Pcdhb19*, *Pfkfb4*, *Pid1*, *Slitrk6*, *Socs3*, *Stox2*, *Tab1*, *Tcf771*, *Trp5*, *Tsc1*, *Zc3h11a*, *Zfp771*, *Dhx30*, *4930519G04Rik*, and *Csnk1a1*. Of these gene candidates, *Socs3* appeared six times within and among the parallel experimental groups, while *Trp53*, *Tsc1*, *Csnk1a1*, and *Nf2* appeared twice. *Nf2*, *Socs3*, *Tcf771*, *Trp53*, *Tsc1*, *Dhx30*, and *Csnk1a1* were previously reported from CRISPR screens based on reporter gene activity (Table S3) [9,10,21,37].

There were two criteria that were used to confirm whether a gene functions in pluripotency exit: cloning efficiency from single cells and passaging capability in differentiation medium [3,20]. We first determined the gene function of our 24 candidate genes by co-transfecting Cas9 and the appropriate sgRNAs into NANOG-GFP mESCs to obtain single-gene homozygous knockout cells (Figures S4 and S5). Single-gene knockout cell lines were constructed using the sgRNAs that were screened from the library or another sgRNA (Table S4). The cell morphology of the targeted cells did not differ significantly from that of wild-type cells (Figure S6A) and the cells grew normally in a complete medium.

Next, we analyzed the expression of *Nanog* in single knockout cell lines. All the cell lines were subjected to flow cytometry to obtain the percentage of GFP+ cells, representing *Nanog* expression. In 23 out of 24 single-knockout cell lines, no significant change in GFP expression was found (Figure 2B), indicating that the single knockout did not affect *Nanog* expression. The sole exception was *Fam24a*-KO cells in which GFP was not expressed and, therefore, not followed up for validation. The remaining 23 single-knockout cell lines showed a significantly higher proportion of GFP+ cells than wild-type cells (which lost considerable GFP expression) following 24 h of culture in N2B27 medium. Of these, *Zpf771*-KO mESCs showed higher GFP expression in the differentiation medium than NANOG-GFP mESCs (Figure 2B).

Most of our candidate genes NANOG-GFP mESCs were able to maintain self-renewal in the N2B27 differentiation medium, and 15 of them were significant, as demonstrated by the validation assay (Figure 2B). These results indicate that the screened genes maintain some ability to sustain self-renewal in differentiation medium, demonstrating the utility of genome-wide PB-CRISPR/Cas9 mESCs screening under differentiation conditions to discover genes that are related to pluripotency exit.

We next interrogated the 23 single-knockout cell line, to assess exit from their stem-ness under differentiation conditions in N2B27. The individual knockout cell lines showed variable capacities for passaging within the differentiation medium. *Nf2*, *Zfp771*, *Olfr769*, *Tsc1*, *Socs3*, *Csnk1a1*, *4930519G04Rik*, and *Trp53*-KO cells could be passaged up to 5, 5, 3, 3, 3, 2, 1, and 11 generations, respectively, while maintaining GFP expression in a proportion of clones (Figure S6B). In the N2B27 differentiation medium, only a certain percentage of the cells maintained both GFP and ESC cellular morphology after several passages. The tolerance of *Nf2*, *Zfp771*, *Olfr769*, *Tsc1*, *Socs3*, *Csnk1a1*, *4930519G04Rik*, and *Trp53*-KO cells to repeat passaging in differentiation medium implies that these genes most likely play an important role in regulating the stem-ness exit of mESCs, supporting previous studies of *Tsc1*, *Trp53*, *Nf2*, *Csnk1a1*, and *Socs3* [10,21,37,38].

**Figure 3:**
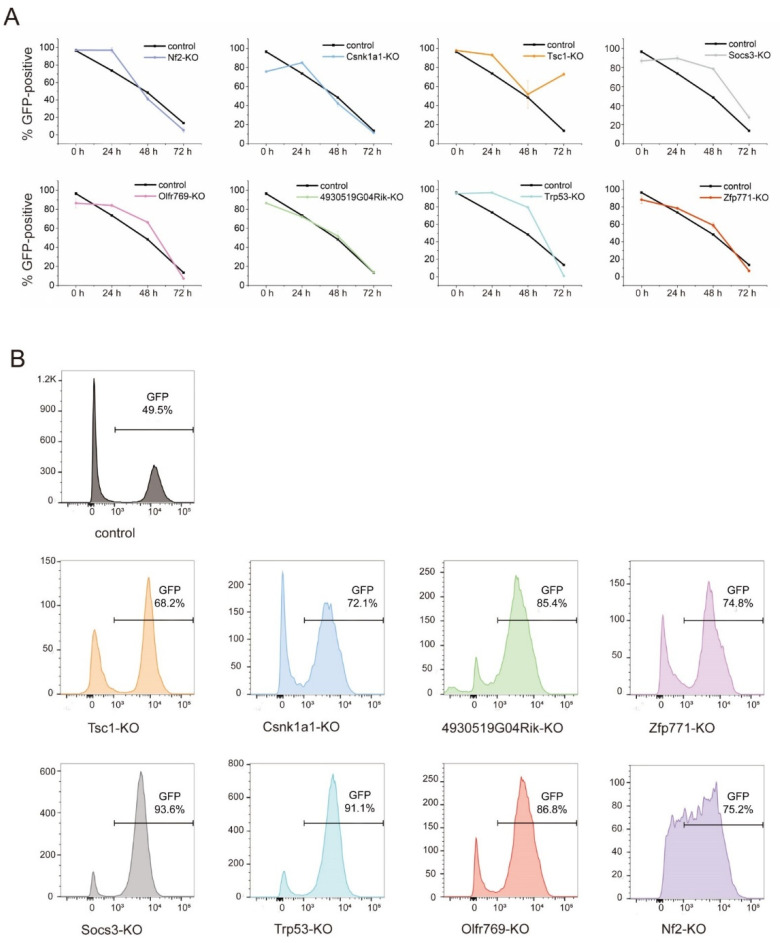
Validation of different candidate genes. (**A**) GFP-positive expression flow sorting results of different candidate genes in differentiation for each time point (0–72 h). The data are shown as the mean ± SEM (*n* = 3 independent experiments). (**B**) GFP expression flow sorting results of different candidate genes at 48 h differentiation. Control was NANOG-GFP mESCs.

Compared to the control group, the loss of GFP in *Nf2*, *Zfp771*, *Olfr769*, *Tsc1*, *Socs3*, *Csnk1a1*, *4930519G04Rik*, and *Trp53*-KO mESCs that were cultured in the N2B27 differentiation medium showed a significant lag-time of up to 72 h (Figure 3A). The GFP expression in *Tsc1*-KO cells even showed a significant upward trend. At 48 h differentiation, the GFP+ cells accounted for 68.3%–93.6% of the total cells in each of these eight KO lines, which were uniformly above that of the control group (Figure 3B and Figure S7). These results indicate that there is an enhancement of the self-renewal network of mESCs in the differentiation medium after gene knockout. Overall, *Nf2*, *Zfp771*, *Olfr769*, *Tsc1*, *Socs3*, *Csnk1a1*, *4930519G04Rik*, and *Trp53*-KO mESCs growing in the 2i/LIF medium had a 3D morphology that was similar to the control mESCs, and were capable of additional limited passage generations while maintaining partial GFP expression in the N2B27 differentiation medium.

**Figure 4:**
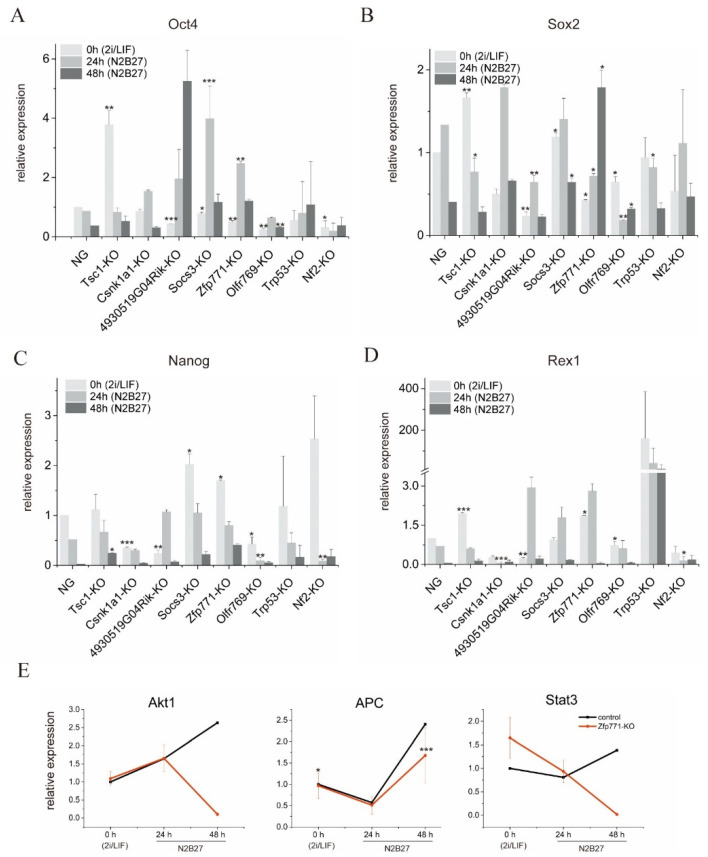
Validation of different candidate genes. (**A**–**D**) Expression kinetics of different pluripotency genes: *Oct4*, *Sox2*, *Nanog*, and *Rex1*, during differentiation of single knockout candidate cell lines. The mean ± SEM are plotted for each time point (*n* = 3 independent experiments). Expression levels were normalized to Gapdh. Student’s *t*-test was performed. * *p* < 0.05, ** *p* < 0.01, *** *p* < 0.001. NG: NANOG-GFP mESCs. (**E**). Kinetics of *Akt1*, *Apc,* and *Stat3* expression in *Zfp771*-KO mESCs during differentiation with 24 and 48 h. The mean ± SEM are plotted for each time point (*n* = 3 independent experiments). Expression levels were normalized to Gapdh. Student’s *t*-test was performed. * *p* < 0.05, ** *p* < 0.01, *** *p* < 0.001. The control was NANOG-GFP mESCs.

To assess the potential functions of *Nf2*, *Zfp771*, *Olfr769*, *Tsc1*, *Socs3*, *Csnk1a1*, *4930519G04Rik*, and *Trp53* with respect to pluripotency exit, we examined gene expression of the pluripotency genes, *Oct4*, *Sox2*, *Nanog*, and *Rex1* in knockout cells at 24 and 48 h of differentiation via RT-qPCR. After 24 h in N2B27 medium, *Nanog* was upregulated in *Tsc1*-KO mESCs; *Sox2* and *Oct4* were upregulated in *Csnk1a1*-KO mESCs; *Oct4* and *Nanog* were upregulated in *4930519G04Rik*-KO mESCs; *Oct4*, *Sox2*, *Nanog*, and *Rex1* were upregulated in *Socs3*-KO; *Oct4*, *Nanog*, and *Rex1* were upregulated in *Zfp771*-KO mESCs; and *Rex1* was upregulated in *Trp53*-KO (Figure 4A–D and Figure S8). After 48 h in N2B27 medium, *Oct4*, *Nanog,* and *Rex1* were upregulated in *Tsc1*-KO mESCs; *Sox2*, *Nanog,* and *Rex1* were upregulated in *Csnk1a1*-KO mESCs; *Oct4*, *Nanog,* and *Rex1* were upregulated in *4930519G04Rik*-KO mESCs; *Oct4*, *Sox2*, *Nanog*, and *Rex1* were upregulated in *Socs3*-KO; *Oct4*, *Nanog*, and *Sox2* were upregulated in *Zfp771*-KO mESCs; *Oct4*, *Nanog,* and *Rex1* were upregulated in *Trp53*-KO; and *Sox2*, *Nanog,* and *Rex1* were upregulated in *Nf2*-KO (Figure 4A–D and S8). The results indicate that the knockout of these candidate genes influenced exit from pluripotency, and the pluripotency genes were able to maintain expression in N2B27 differentiation medium.

The above results indicate that knockout of the validated genes can prompt cell pluripotency maintenance to some extent in N2B27 differentiation medium. The effects of different gene knockouts on pluripotency gene expression varied, indicating that our eight candidates act on different pathways. Among the eight genes that were validated, *Zfp771* showed a high expression of pluripotency genes. Moreover, its pluripotent gene expression profile was very similar to that of the reported gene *Socs3*. Therefore, we further analyzed *Zpf771* function with respect to pluripotency exit.

### 3.3. Zfp771 Function Is Required for Exit from Pluripotency

Of all the genes we screened, *Zfp771* was present across different screening groups, and *Zfp771* knockout demonstrated the best pluripotency maintenance in the differentiation medium, suggesting that *Zfp771* is likely to play an important role in regulating pluripotency exit of mESCs.

We further examined the expression of *Stat3*, *Akt1*, and *Apc* in *Zfp771*-KO cell lines. *Stat3* was upregulated in the 2i/LIF medium. After 48 h in N2B27 medium, *Stat3, Akt1,* and *Apc* were upregulated in the control mESCs. In comparison, *Stat3* and *Akt1* were downregulated and *Apc* was upregulated in *Zfp771*-KO mESCs (Figure 4E). These results suggest that *Zfp771* may play a role in the LIF-STAT3 pathway.

**Figure 5:**
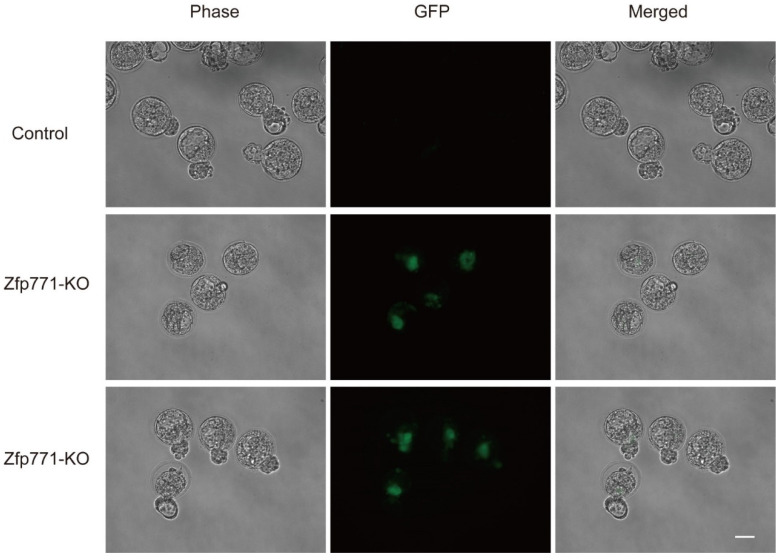
Zfp771-KO mESCs in blastocyst experiments. Knockout of Zfp771 by expression of NANOG-GFP mESCs in blastocyst experiments (*n* = 2 technical replicates of cells). The control was NANOG-GFP mESCs. Scale bar, 100 µm.

We also verified the development of *Zfp771*-KO cells in embryos by injecting *Zfp771* single-knockout cells with GFP reporter into 8-cell stage embryos and observing that GFP was expressed up to the blastocyst stage (Figure 5). The control cells were injected with NANOG-GFP mESCs without knockouts. This demonstrates that the lack of *Zfp771* in blastocysts can hinder cell exit from pluripotency.

### 3.4. Zfp771-KO Cell Transcriptomes Reveal Differences in Pluripotency Gene Expression

**Figure 6:**
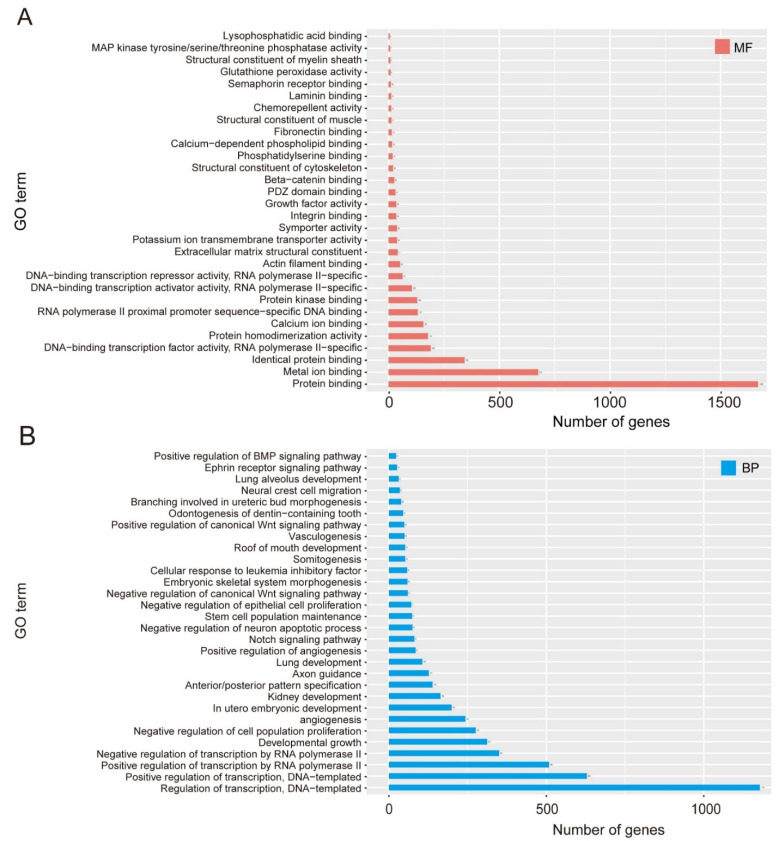
Transcriptome profile in Zfp771-KO mESCs. (**A**) The bar chart shows the names of the top30 GO TERMs and the corresponding number of genes. According to the classification of GO, Class is divided into: MF (molecular function) by default (*n* = 3 independent experiments). (**B**) The bar chart shows the names of the top30 GO TERM and the corresponding number of genes. According to the classification of GO, Class is divided into: BP (biological process) by default (*n* = 3 independent experiments). The GO enrichment analysis of significantly different genes was performed by selecting different genes with an FDR that was less than 0.05 to do GO enrichment.

**Figure 7:**
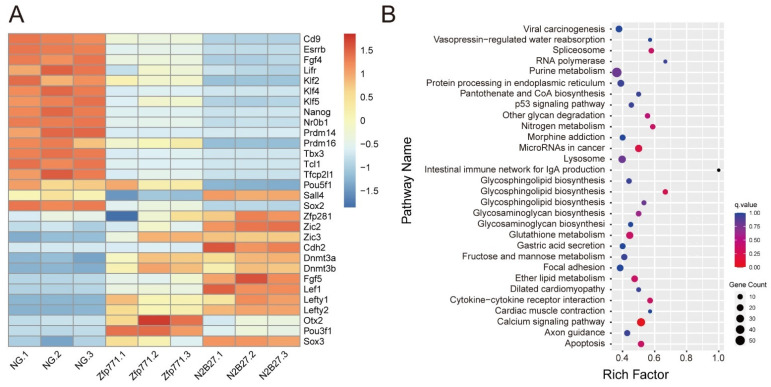
Transcriptome profile in Zfp771-KO mESCs. (**A**) Expression profile of different pluripotency period marker genes (*n* = 3 independent experiments). The control was NANOG-GFP mESCs. (**B**) Distribution of KEGG functional annotations of Zfp771-KO after comparison with mESCs and differentiated mESCs for significantly differentially expressed genes. Significant gene pathway enrichment analysis was performed by selecting differentially expressed genes with FDR that was less than 0.05 to do KEGG enrichment. The scatter plot is a graphical presentation of the results of the KEGG enrichment analysis. In this plot, the degree of KEGG enrichment is measured by the RichFactor, *p*-value, and the number of genes that were enriched to this pathway. The RichFactor is the ratio of the number of differentially expressed genes that are enriched in the pathway to the total number of genes in the pathway, with a larger RichFactor indicating greater enrichment with a q-value that is close to zero indicating more significant enrichment. We selected the 30 most significantly enriched pathway entries for display in this figure, or all of them if there are less than 30 enriched pathway entries (*n* = 3 independent experiments).

RNA-seq analysis was performed to further investigate the effect of *Zfp771* on pluripotency exit of mESCs (Figure 6 and Figure 7, Table S5). RNA expression in *Zfp771*-KO cells was differentially up-regulated and down-regulated for mESCs control and versus N2B27 control (Figure S8). Transcriptomes were compared between ES cells in N2B27 differentiation medium (120 h), ES cells in 2i/LIF medium, and *Zfp771*-KO mESCs in N2B27 differentiation medium. Enrichment of MAPK phosphorylation was observed through GO analysis when *Zfp771*-KO cells were compared with normal ESCs (Figure 6A). In contrast, when compared with the transcriptome of differentiated cells, enrichment was observed in the positive regulators of the BMP signaling pathway, positive and negative regulators of the WNT pathway, Notch signaling pathway, and the stem cell population maintenance pathway (Figure 6B). This indicates that knockout of *Zfp771* has a positive effect on the maintenance of cell stemness in N2B27 differentiation medium.

We further compared the specific expression of pluripotency and formative genes [10] in normal mESCs and differentiated mESCs with *Zfp771*-KO cells (Figure 7). The expression of pluripotency genes was up-regulated in *Zfp771*-KO cell lines whereas the expression of differentiated genes decreased. These results are consistent with the screening results and indicate that *Zfp771* plays a negative regulatory role in pluripotency exit.

## 4. Discussion

We performed a genome-wide screen for genes leading to early differentiation of mESCs using the CRISPR/Cas knockout screening system in a strict N2B27 differentiation culture system without small molecule inhibitors, LIF, and feeder layer cells. We identified a series of known and unknown genes that affect the self-renewal maintenance of mESCs.

Some genes from our screen, such as *Socs3* and *Tsc1*, have been validated in previous studies and are very relevant for the early differentiation of mESCs. *Socs3* is regulated by the JAK/STAT signaling pathway by binding to gp130 and other cytokines. *Socs3* suppresses JAK/STAT signaling in two ways. First, *Socs3* can directly inhibit the catalytic activity of JAK1, JAK2, or TYK2. Second, Socs3 recruit’s proteins to produce E3 ligase, ubiquitinating JAK, and cytokine receptors, making it a target for proteasome degradation [39]. *Tsc1* and *Tsc2* act together, and TSC1, TSC2, and TBC1D7 form the TSC complex. The TSC complex binds to and activates mTORC1, which in turn affects the AKT-MAPK pathway. The upstream WNT pathway activates mTORC1 by inhibiting TSC1 [40]. These studies, together with the above results, support the validity of our screening.

We also identified genes that were closely related to the early differentiation of ESCs, such as *Trp53*, *Nf2*, and *Csnk1a1*. *Trp53* activation via the mTORC1 pathway is regulated by p38 and JUNK1 from the MAPK pathway, among others [41,42]. The role of the JNK/JUN pathway as a negative regulator of the p53 tumor suppressor is also supported by the oncogenic role of activated JNK in tumor models, which is influenced by the conditional JNK1 allele in the JNK pathway [41]. The FGF-MAPK signaling pathway is important for maintaining pluripotency of stem cells [41]. The JNK and p38 MAPK pathways are two important MAPK pathways. *Trp53* is regulated by these two pathways, which means that it is regulated by p38 and JUNK1 via the MAPK pathway through the activation of the mTORC1 pathway [41,42].

*Csnk1a1* encodes subunits that form casein kinase Ia, a key regulator of the WNT signaling pathway, which is a component of the β-catenin-destruction complex 1. It has been shown that *Csnk1a1* is a kinase that fulfills all the criteria for a key mediator of the genotoxic stress-induced WNT signaling pathway, antagonizing p53-mediated apoptosis in ES cells [43,44]. Therefore, these genes may play an important role in maintaining the pluripotent state of mESCs.

*Nf2* is a tumor suppressor gene that encodes Merlin. Merlin is a multifunctional protein that is involved in the regulation of intracellular and extracellular signaling pathways with roles in cell fate, shape, proliferation, survival, and motility [45]. It has been shown that PI3K expression is inhibited in Merlin-deficient mouse Schwann cells and cell proliferation is selectively reduced by caspase-dependent apoptosis causing varying degrees of cell death [46]. Whether *Nf2* plays the same role in stem cells has not been determined.

Additionally, our screen revealed previously unreported genes: *Olfr769*, *Zpf771*, and *4930519G04Rik* as candidate factors affecting mESCs pluripotency maintenance, of which *Zfp771*-KO mESCs were examined in detail. To our knowledge, this is the first study to report the functionality of *Zfp771*. We found no change in cell morphology after *Zfp771* knockout in mESCs (Figure S5). *Zfp771*-KO mESCs showed a lag of *Nanog* expression (GFP) loss in differentiation medium at different times (Figure 2 and Figure 3). The pluripotency genes of *Zfp771*-KO cells expression were up-regulated in N2B27 differentiation medium (Figure 4). *Zfp771*-KO mESCs showed differentiation block in vitro blastocyst experiments (Figure 5). We speculate that *Zfp771* may function as a transcriptional repressor. Through direct or indirect links to *Nanog* transcriptional regulation, *Zfp771* may affect the state of self-renewal. *Olfr769* is a family member of olfactory receptor genes. Embryonic stem cell differentiation begins with ectodermal development toward the nervous system, and the ablation of olfactory receptors may be presumed to prevent proper olfactory neural development, thus helping to maintain stem cells in a pluripotent state. However, the exact mechanism needs to be investigated in the future.

Our screen found genes that are both known and unknown with respect to their role in pluripotency exit. Future work will require full-scale validation of the candidate factors that were obtained from our study. In addition, exploration of these genes’ roles in the differentiation of mESCs and the mechanisms of interaction with other genes such as *Olfr769*, *Nf2*, *Trp53*, *Zpf771*, *4930519G04Rik*, and *Csnk1a1* is warranted. Thus, our screening results provide novel gene candidates as well as a novel protocol to better understand the molecular mechanisms of exit from pluripotency.

The very strict differentiation screening conditions that we used played a key role throughout our study and underlies the low number of revealed candidates compared to previous studies. In the future, by using different reporter systems (e.g., Oct4-GFP reporter) for screening under strict differentiation culture conditions, we should be able to reveal more regulators of pluripotency exit. In addition to the mouse, the cell culture systems of other large animals, especially human pluripotent stem cells, can be further optimized for use with our stringent screening methods to characterize crucial stem cell genetic programs.

## 5. Conclusions

We successfully performed a stringent screen with a genome-wide CRISPR knockout library for the key genes controlling the exit from pluripotency. The candidate genes *Socs3*, *Tsc1*, *Trp53*, *Nf2*, *Csnk1a1*, *Zpf771*, and *Olfr769* were verified to be associated with the regulatory network for pluripotency exit. Further, the transcriptome analysis and experiments of *Zfp771*-KO mESCs demonstrated that *Zfp771* affects the exit of mESCs pluripotency through the LIF pathway. These findings provide a new paradigm for genome-wide screening, and more accurate screening results can be obtained by strict screening conditions. They can also be applied to the screening of problems that are related to human or large animal embryonic stem cells.

## Data Availability

The datasets generated during and/or analyzed during the current study are available from the corresponding author on reasonable request.

## Supplementary Materials

The following supporting information can be downloaded at: https://www.mdpi.com/article/10.3390/cells11152289/s1, Figure S1. NANOG-GFP screening reporter. Figure S2. Copy number detection in cell clones that were obtained by screening. Figure S3. Screening to obtain cells for relevant pluripotency assays. Figure S4. Generation of mESCs clones at the target loci. Figure S5. Generation of mESCs clones at the target loci. Figure S6. Construction of candidate single knockout cell lines. Figure S7. Validation of different candidate genes. Figure S8. Validation of different candidate genes. Table S1. The primers for RT-qPCR. Table S2. Five groups of screening results. Table S3. The list of candidate genes. Table S4. The sgRNAs of candidate genes. Table S5. RNA-seq results of *Zfp771*-KO mESCs.

## Abbreviations

ESCs	Embryonic stem cells
mESCs	Mouse embryonic stem cells
PB	piggyBac
GFP	Green fluorescent protein
LIF	Leukemia inhibitory factor
2i	2 inhibitors (CHIR99021, PD0325901)
KO	Knockout
cDNA	Complementary DNA
qPCR	Quantitative RT-PCR
GFP+	GFP-positive
GO	Gene Ontology
