# Supplementary material for: A Genome-Wide CRISPR Screen Identifies Factors Regulating Pluripotency Exit in Mouse Embryonic Stem Cells
Source: Cells. 2022 Jul 25;11(15):2289. doi: 10.3390/cells11152289 (PMC9331787; doi:10.3390/cells11152289)
Supplement: Supplementary file 1 [file cells-11-02289-s001.zip › Figure S5.pdf]

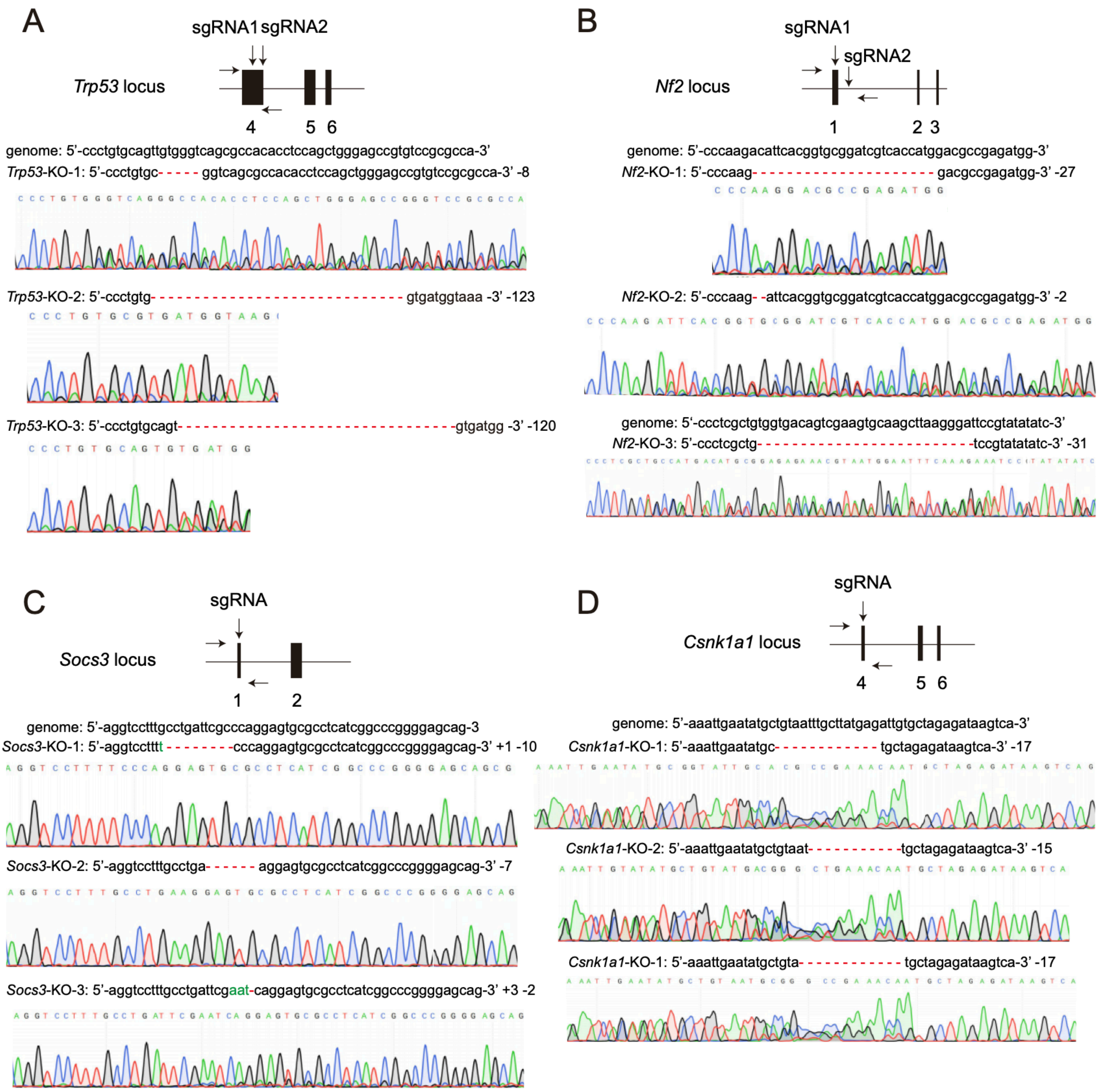
A
sgRNA1 sgRNA2
Trp53 locus
4
5 6
genome: 5'-ccctgtgcagttgtgggtcagcgccacacctccagctgggagccgtgtccgcgcca-3'
Trp53-KO-1: 5'-ccctgtgc- - - - - ggtcagcgccacacctccagctgggagccgtgtccgcgcca-3' -8
Trp53-KO-2: 5'-ccctgtg- gtgatggtaaa -3' -123
Trp53-KO-3: 5'-ccctgtgcagt- -gtgatgg -3' -120
B
sgRNA1
sgRNA2
Nf2 locus
1
2 3
genome: 5'-cccaagacattcacggtgcggatcgtcaccatggacgccgagatgg-3'
Nf2-KO-1: 5'-cccaag- gacgccgagatgg-3' -27
Nf2-KO-2: 5'-cccaag- -attcacggtgcggatcgtcaccatggacgccgagatgg-3' -2
genome: 5'-ccctcgctgtggtgacagtcgaagtgcaagcttaagggattccgtatatatc-3'
Nf2-KO-3: 5'-ccctcgctg- -tccgtatatatc-3' -31
C
sgRNA
Socs3 locus
1
2
genome: 5'-aggtcctttgcctgattcgcccaggagtgcgcctcatcggcccggggagcag-3
Socs3-KO-1: 5'-aggtcctttt- - - - - - - -cccaggagtgcgcctcatcggcccggggagcag-3' +1 -10
Socs3-KO-2: 5'-aggtcctttgcctga- - - - - - aggagtgcgcctcatcggcccggggagcag-3' -7
Socs3-KO-3: 5'-aggtcctttgcctgattcgaat-caggagtgcgcctcatcggcccggggagcag-3' +3 -2
D
sgRNA
Csnk1a1 locus
4
5 6
genome: 5'-aaattgaatatgctgtaatttgcttatgagattgtgctagagataagtca-3'
Csnk1a1-KO-1: 5'-aaattgaatatgc- - - - - - - - - - - - tgctagagataagtca-3' -17
Csnk1a1-KO-2: 5'-aaattgaatatgctgtaat- - - - - - - - - - -tgctagagataagtca-3' -15
Csnk1a1-KO-1: 5'-aaattgaatatgctgta- - - - - - - - - - - - tgctagagataagtca-3' -17
