# Supplementary material for: A Genome-Wide CRISPR Screen Identifies Factors Regulating Pluripotency Exit in Mouse Embryonic Stem Cells
Source: Cells. 2022 Jul 25;11(15):2289. doi: 10.3390/cells11152289 (PMC9331787; doi:10.3390/cells11152289)
Supplement: Supplementary file 1 [file cells-11-02289-s001.zip › Supplemental Figures legends.pdf]

Figure S1. NANOG-GFP screening reporter.

A. Schematic diagram of vectors associated with PB-CRISPR/Cas9 gene targeting system. Library plasmid vector and Cas9 protein expression vector.

B. Isolate of a mouse embryonic stem cell line with Nanog-GFP reporter. The top row of the figure shows mouse blastocysts containing the Nanog-GFP reporter, the morphology of which is shown in the left panel under bright field conditions, and the results observed under fluorescence conditions are shown in the right panel, with the blastocysts expressing green fluorescent protein. The bottom row of the figure shows embryonic stem cells obtained from the blastocysts after passaged amplification, the morphology of which is shown in the left panel under bright field conditions, and the results observed under fluorescence conditions are shown in the right panel, with the cells expressing green fluorescent protein, and the scale bar is 50 μm.

C. Identification of mESCs expressing the Nanog-GFP reporter for Cas9 protein. Western Blot assay results of Cas9 expression in the reporter cells, with the top row showing the Flag-tagged Cas9 protein expression assay and the bottom row showing the assay of internal reference Tubulin expression, with ES-Cas9 as a positive control and WT as a negative control.

D. Differentiation process of Nanog-GFP reporter cells in the N2B27 differentiation medium. The scale bar is 500 μm.

E and F. Results of differentiation medium screening to obtain pluripotent cells.

E. The top row shows the morphology of the mixed cells obtained by differentiation medium screening. The top row shows the morphology of the cells under bright field conditions, with some cells differentiated to a feeder-like state and some cells in stereoclonal aggregates with full morphology and clear edges; the bottom row shows the GFP expression under fluorescence irradiation, with some cells positive. The scale of 50 μm. F. The proportion of green cells in the mixed cells obtained from 3 groups of different screens was sorted using flow cytometry analyzer.

Figure S2. Copy number detection in cell clones obtained by screening.

A and B. Identification of sgRNA copy number in monoclonal clones of pluripotent cells obtained by screening. A. Specific design strategy for Southern Blot identification. The probes were designed in the region near the hU6 promoter (indicated by arrows), and the black horizontal line outside the PB junction is the genome, as the vector contains one HindIII restriction endonuclease site, and each band ≥1.9 kb of genomic DNA after HindIII digestion for subsequent reactions indicates one copy number. B. Results of sgRNA integration into the genome of 10 cell lines of Clone 1-Clone 10 identified by Southern Blot, the number of integrated copies of sgRNA in the cells was 1-5.

C. Statistics of sgRNA TA cloning results in the monoclonal clones of pluripotent cells obtained by screening. The horizontal coordinate represents the cell clone number, the vertical coordinate represents the number of sgRNAs in the 10 monoclonal cells analyzed by sequencing using the TA cloning method, and the content marked on the blue bar graph represents the types of sgRNAs detected in the 10 monoclonal cells.

Figure S3. Screening to obtain cells for relevant pluripotency assays.

A. Immunofluorescence detection of pluripotent proteins in mixed cells obtained by screening. The scale bar is 50 μm.

B. Results of karyotype analysis of mixed cells obtained by screening (n = 3 independent cells).

C. Results of screening for negative regulators of pluripotency. The overlapping set of genes measured by 5 sets of data was plotted. 1260, 388, 697, 82, and 95 genes were measured by 5 sets of data for FACS-1, FACS-2, FACS-3, Puro-1, and Puro-2, respectively, and 34 identical genes were screened for the 5 sets of data.

Figure S4. Generation of mESCs clones at the target loci.

Sequencing confirmation of gene target locus. Genome sequences are shown in black, insertions, or mismatches are shown in green, deletions are shown in red dashed lines (n = 3 independent homozygous cells). Numbers show the number of deletions and additions of bases.

Figure S5. Generation of mESCs clones at the target loci.

Sequencing confirmation of gene target locus. Genome sequences are shown in black, insertions, or mismatches are shown in green, deletions are shown in red dashed lines (n = 3 independent homozygous cells). Numbers show the number of deletions and additions of bases.

Figure S6. Construction of candidate single knockout cell lines.

A. Cell morphology and GFP expression of candidate gene single knockout cell lines in 2i medium. The scale bar is 100 μm.

B. Single knockout cell line passaged in N2B27 differentiation medium. The scale bar is 20 μm.

Figure S7. Validation of different candidate genes.

GFP expression flow sorting results of different candidate genes in a differentiation 48 h.

Figure S8. Validation of different candidate genes.

A–D. Expression kinetics of different pluripo-tency genes: *Oct4*, *Sox2*, *Nanog*, and *Rex1*, during the differentiation of single knockout candidate cell lines. Mean and SEM are plotted for each time point (n = 3 independent experiment). Expression levels were normalized to Gapdh. Mean and SEM are plotted for each time point (n = 3 independent experiment). Student's t test was performed. $*p < 0.05$, $**p < 0.01$, $***p < 0.001$. E. Transcriptome profile in Zfp771-KO mESCs. Expression changes of differential genes in Zfp771-KO with mESC and N2B27 as controls.
