# Supplementary figures and images for: A Genome-Wide CRISPR Screen Identifies Factors Regulating Pluripotency Exit in Mouse Embryonic Stem Cells
Source: Cells. 2022 Jul 25;11(15):2289. doi: 10.3390/cells11152289 (PMC9331787; doi:10.3390/cells11152289)

A

PB-sgRNA library

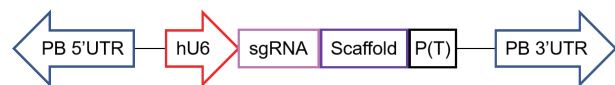

PB-CRISPR/Cas9 W9

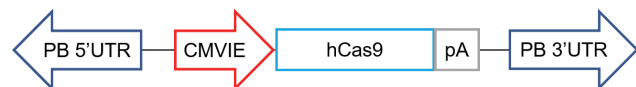

B

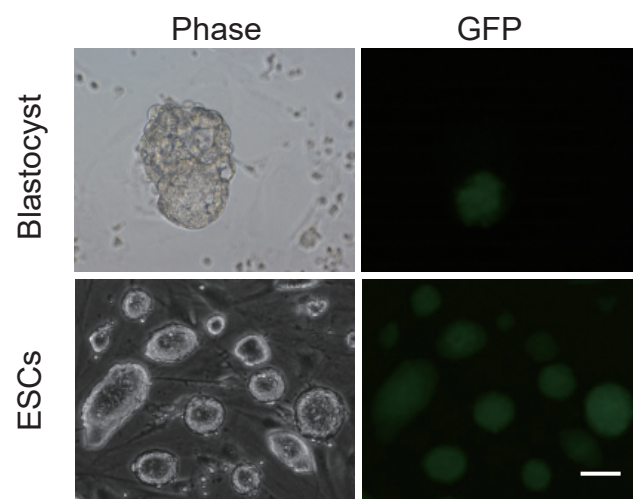

C

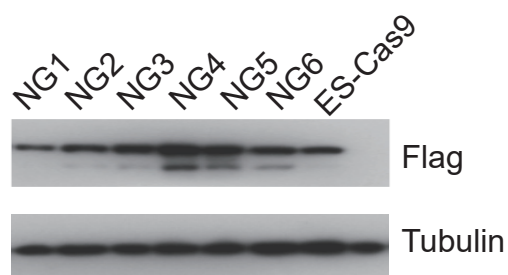

D

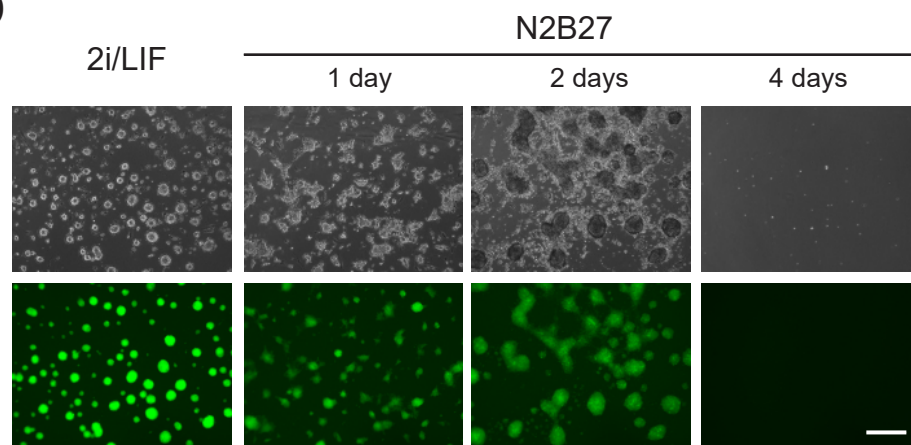

E

Puro-1

Puro-2

Phase

GFP

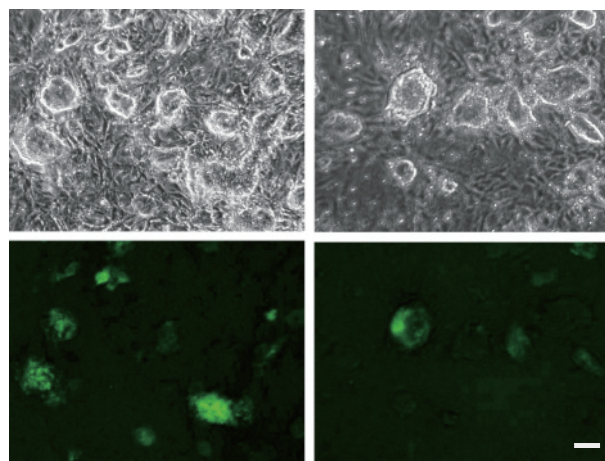

F

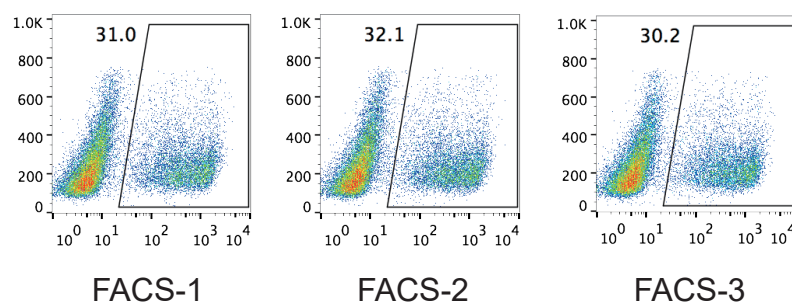

Supplement: Supplementary file 1 [file cells-11-02289-s001.zip › Figure S1.pdf]

A

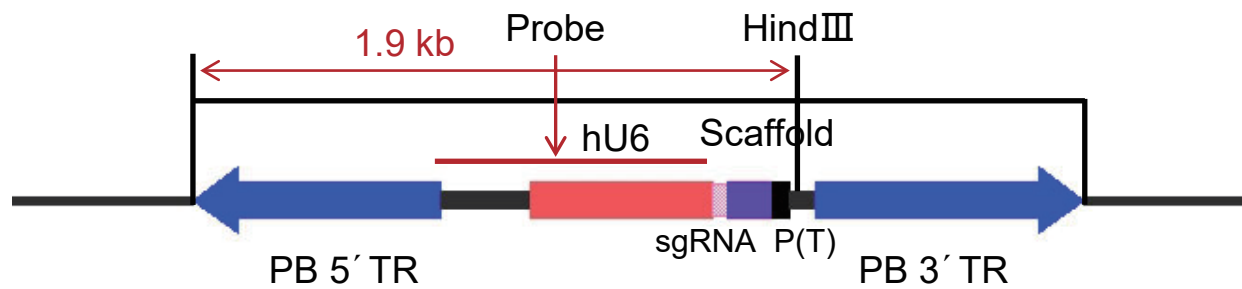

B

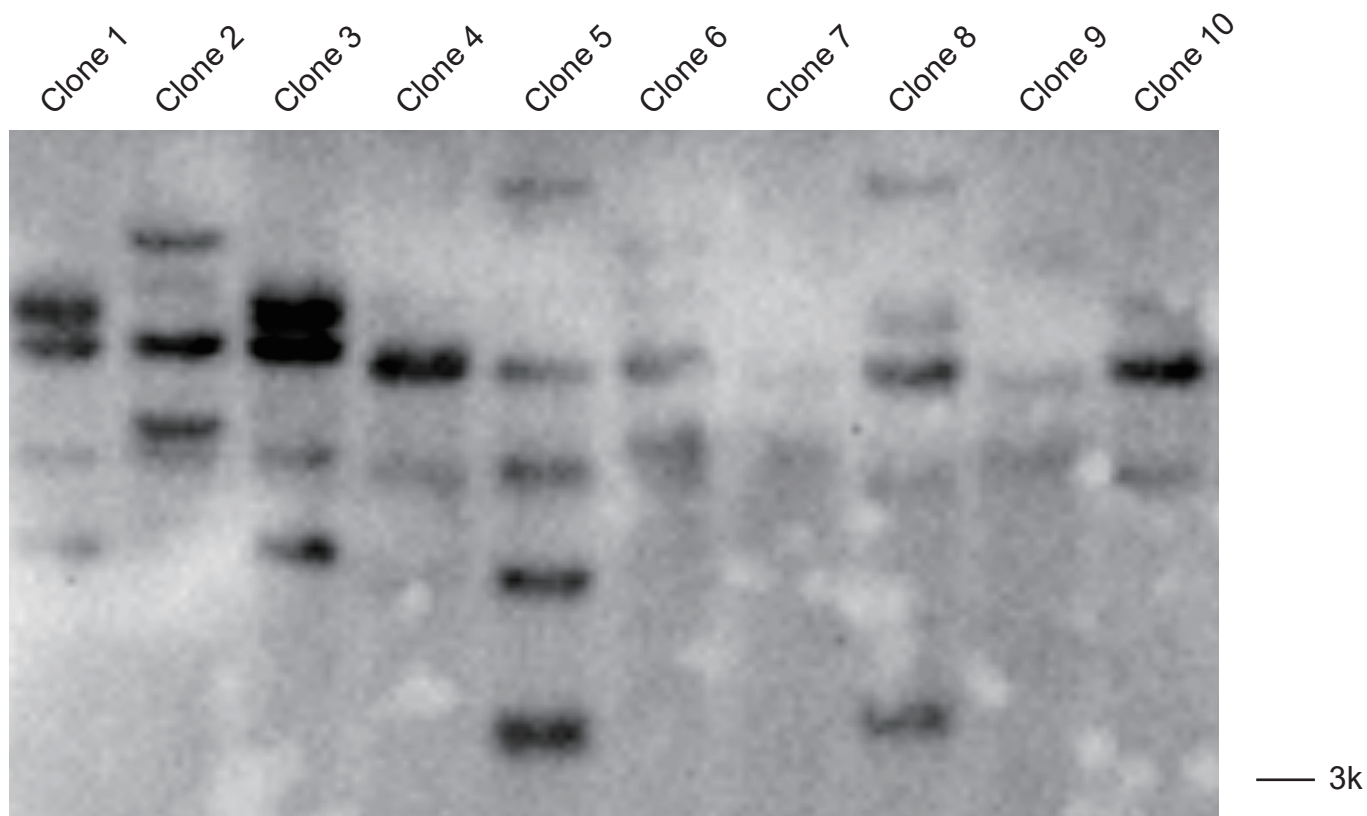

C

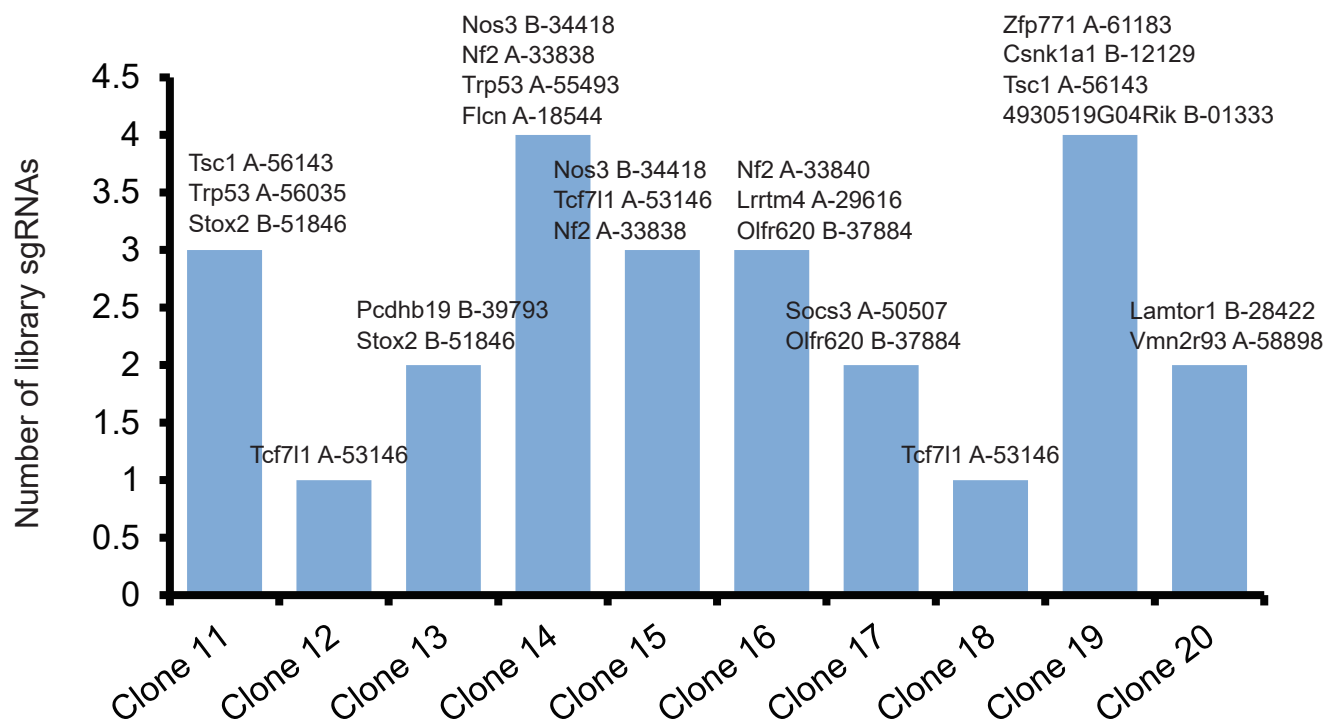

Supplement: Supplementary file 1 [file cells-11-02289-s001.zip › Figure S2.pdf]

A

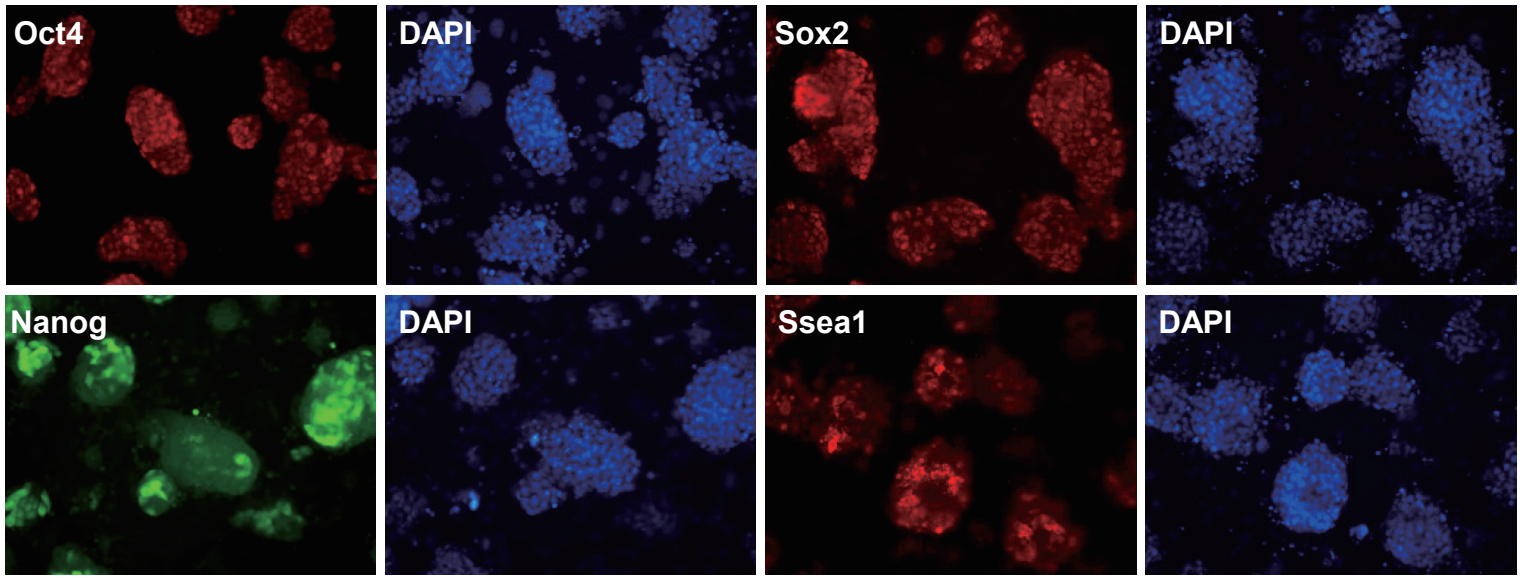

B

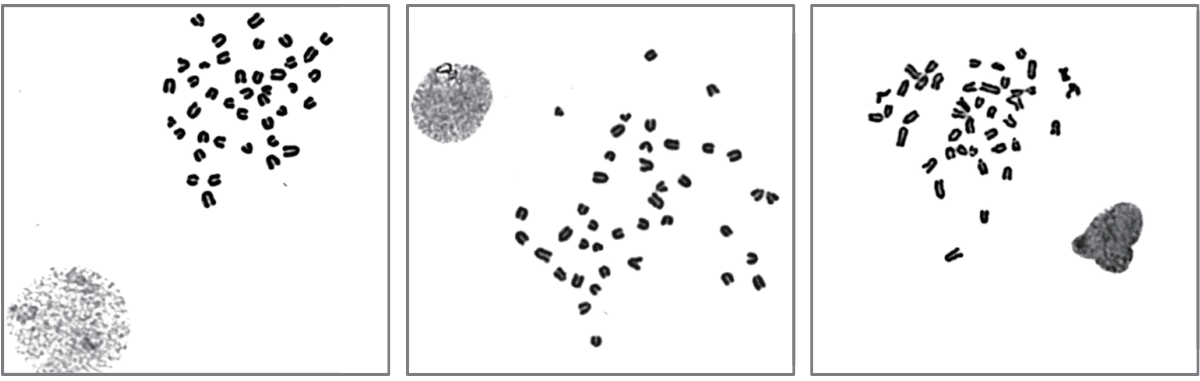

C

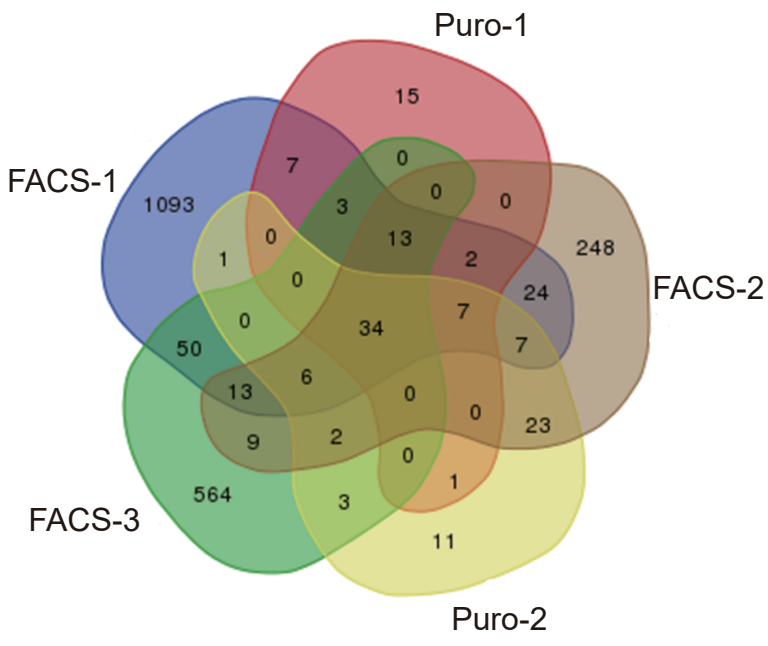

Supplement: Supplementary file 1 [file cells-11-02289-s001.zip › Figure S3.pdf]

**A**

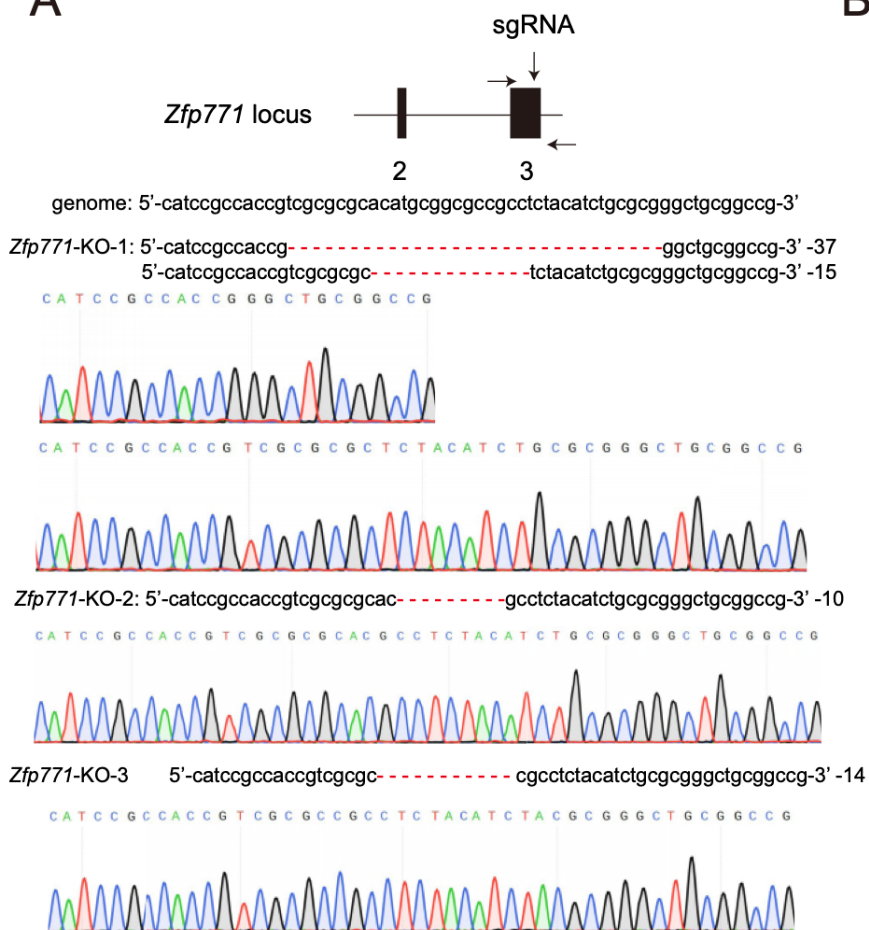

**B**

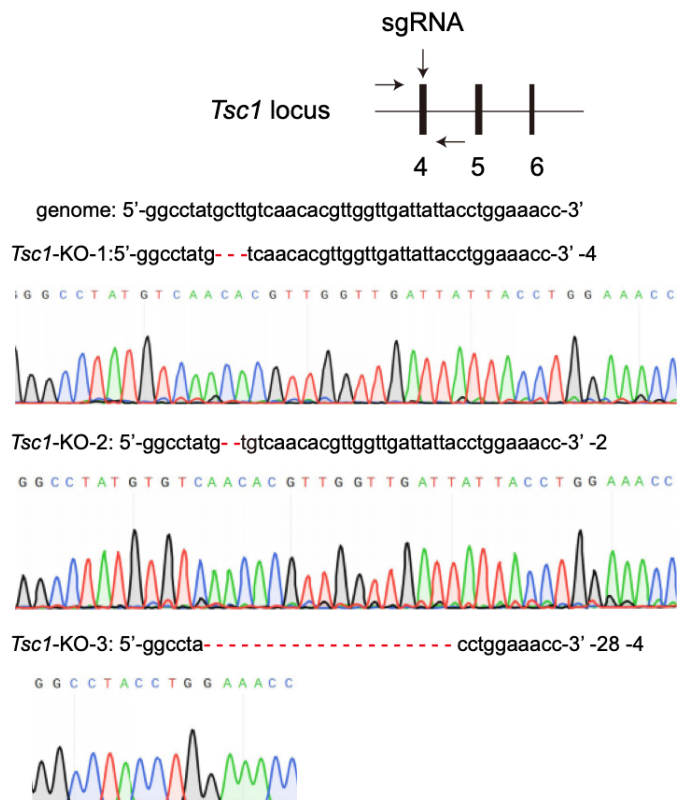

**C**

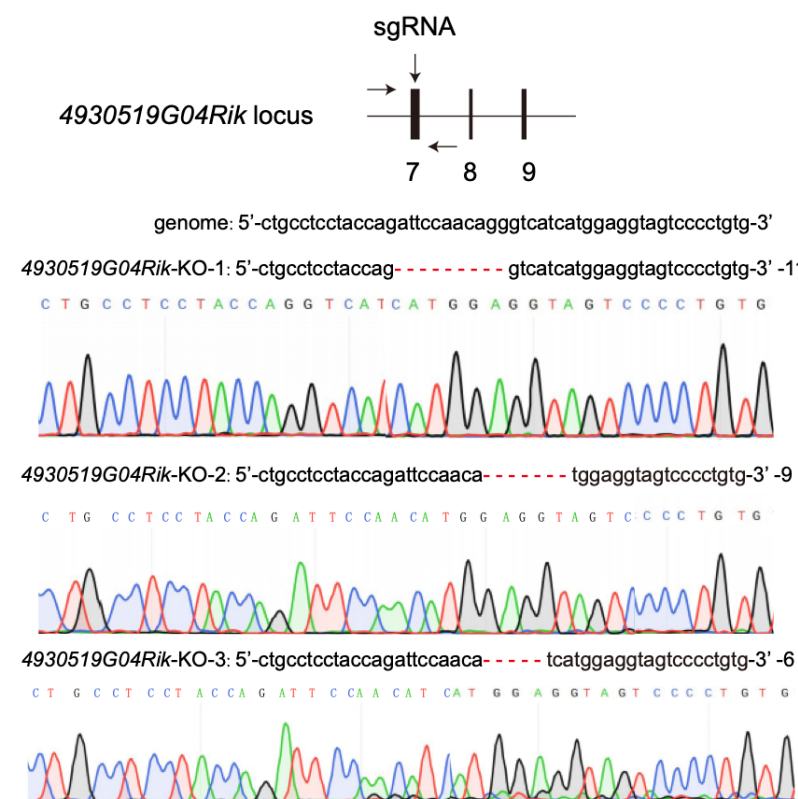

**D**

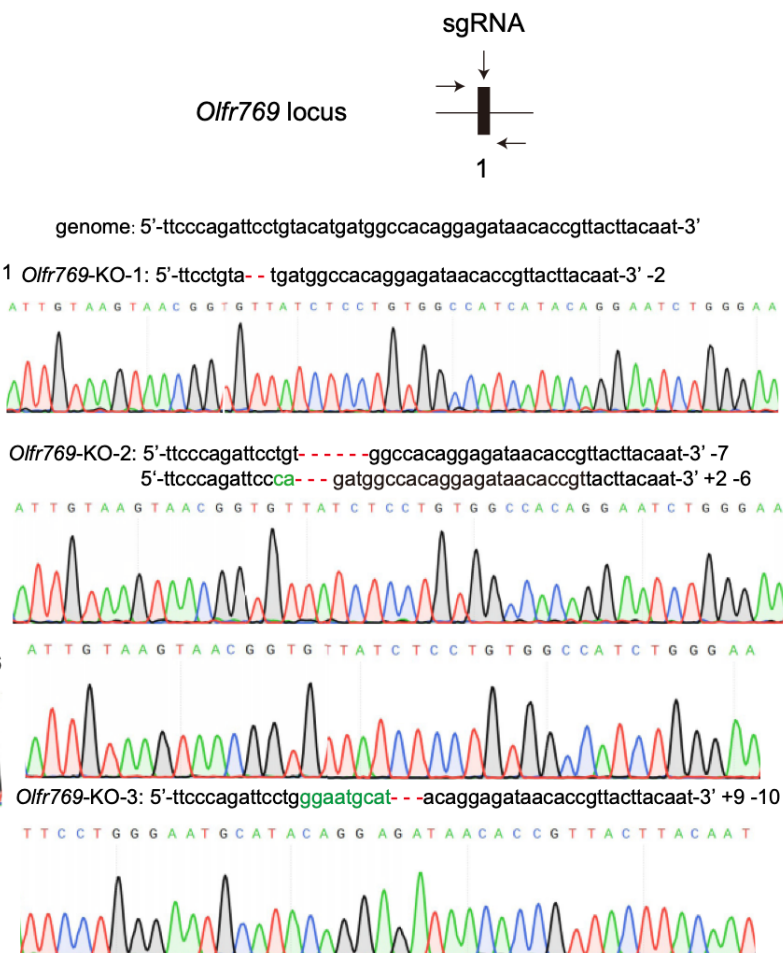

Supplement: Supplementary file 1 [file cells-11-02289-s001.zip › Figure S4.pdf]

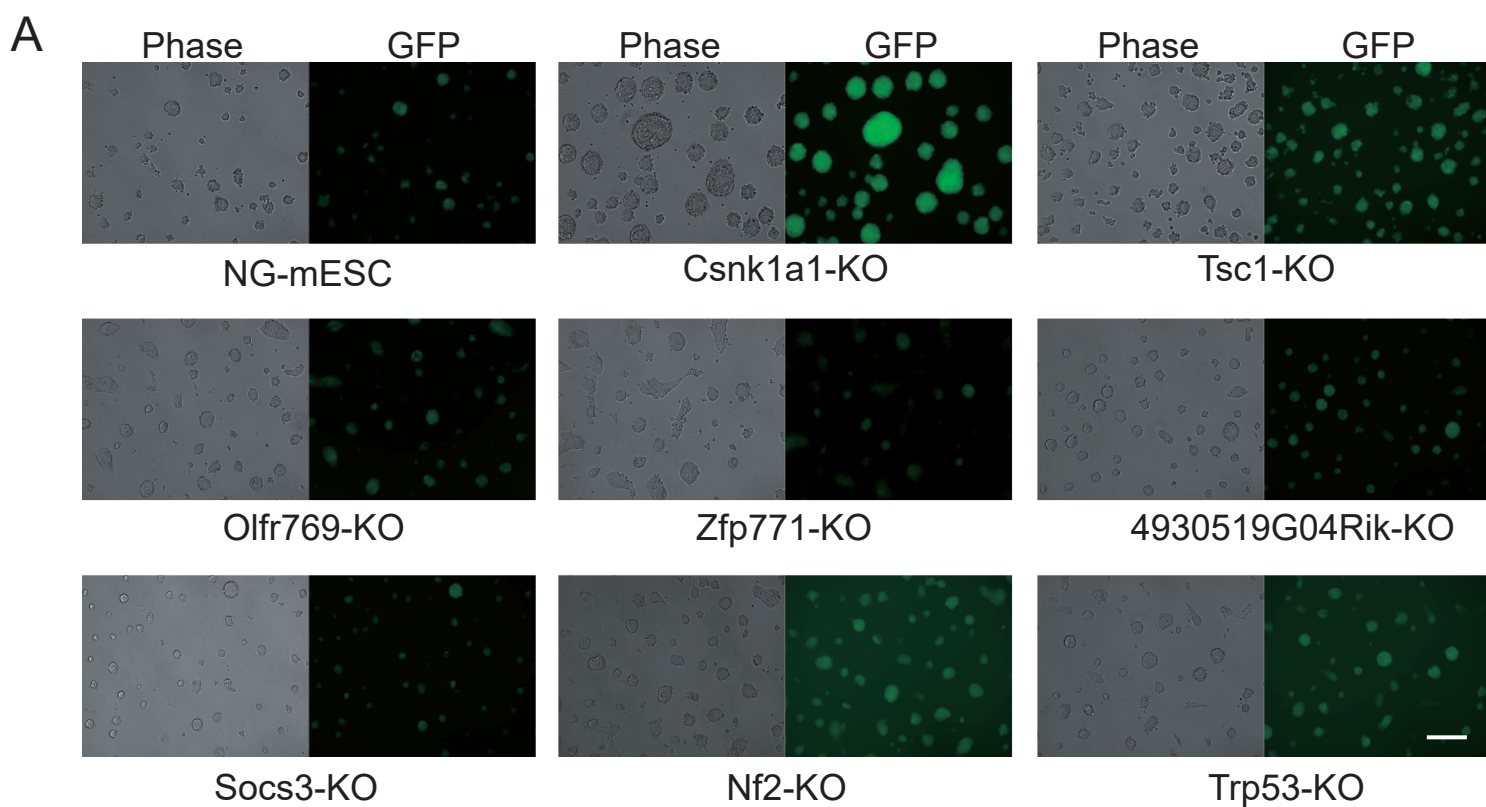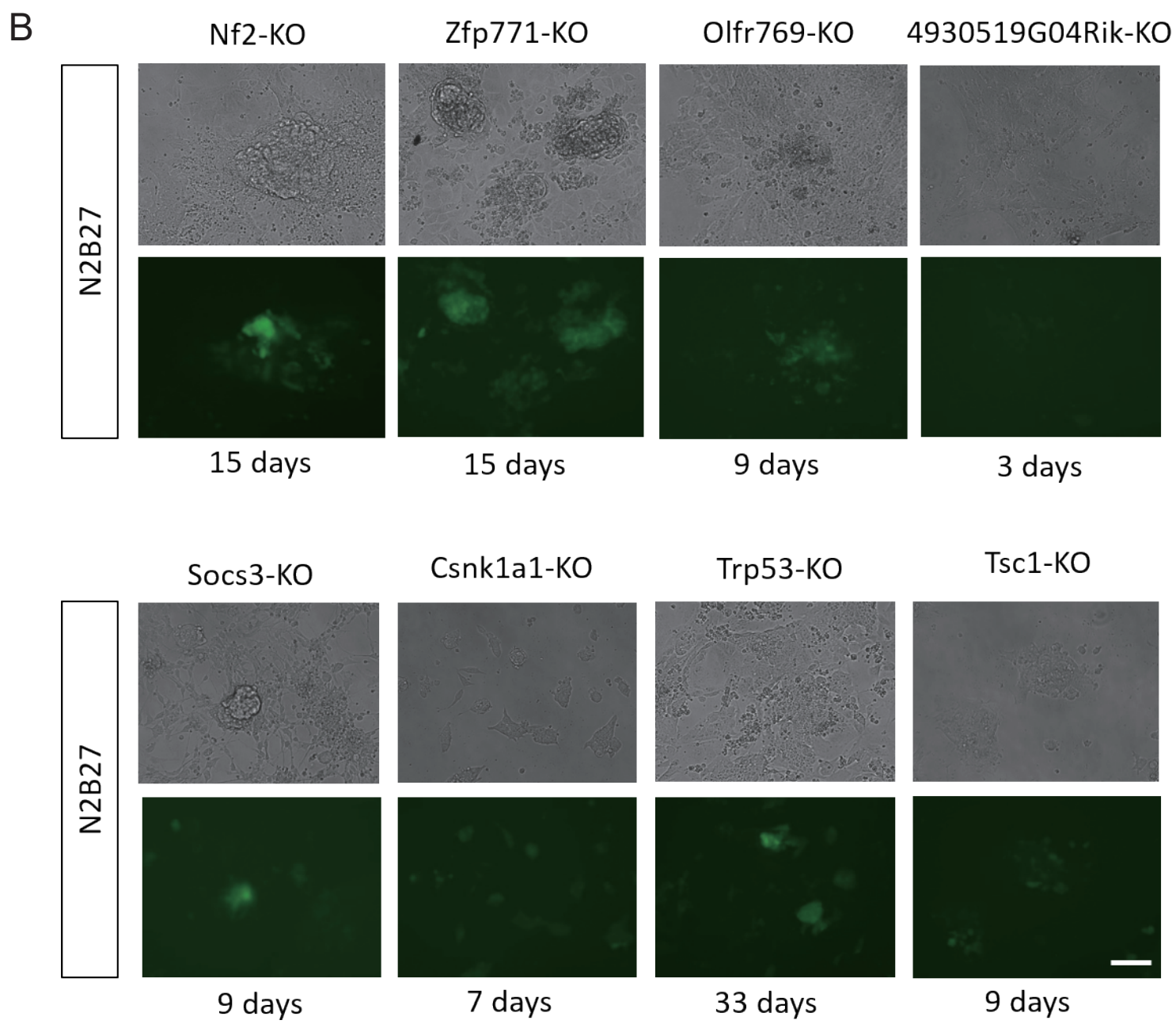

Supplement: Supplementary file 1 [file cells-11-02289-s001.zip › Figure S6.pdf]

mESCs

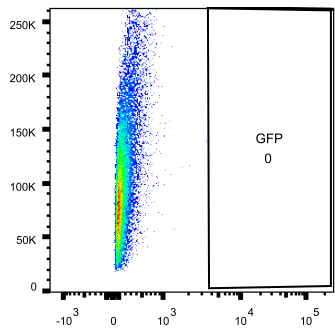

control

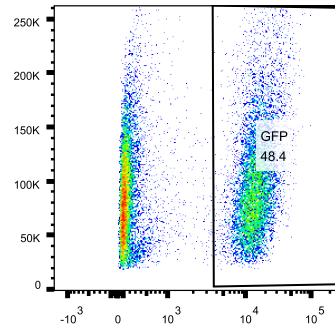

Tsc1-KO

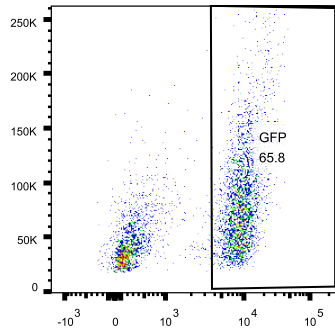

Csnk1a1-KO

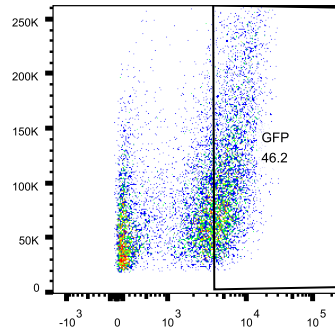

4930519G04Rik-KO

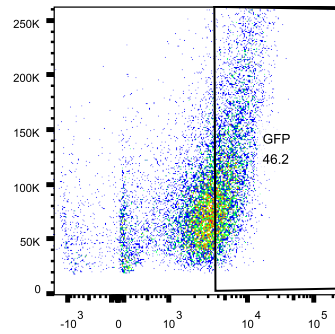

Zfp771-KO

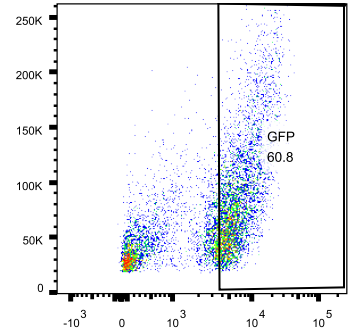

Socs3-KO

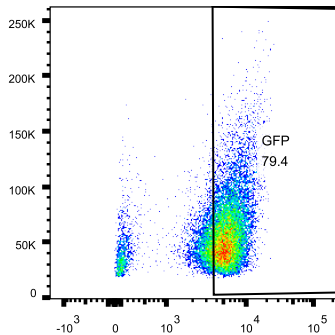

Trp53-KO

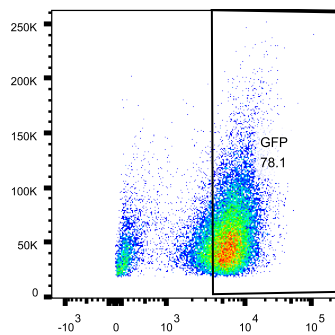

Olf769-KO

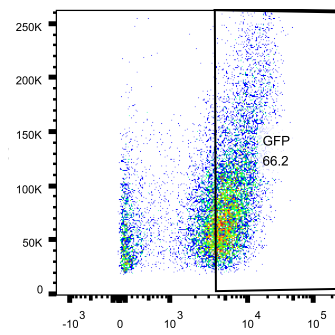

Nf2-KO

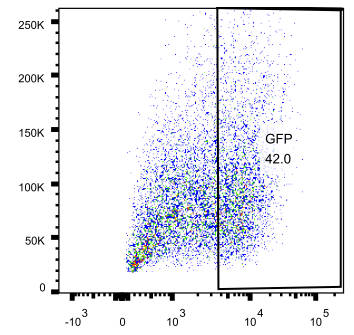

FSC

GFP

Supplement: Supplementary file 1 [file cells-11-02289-s001.zip › Figure S7.pdf]

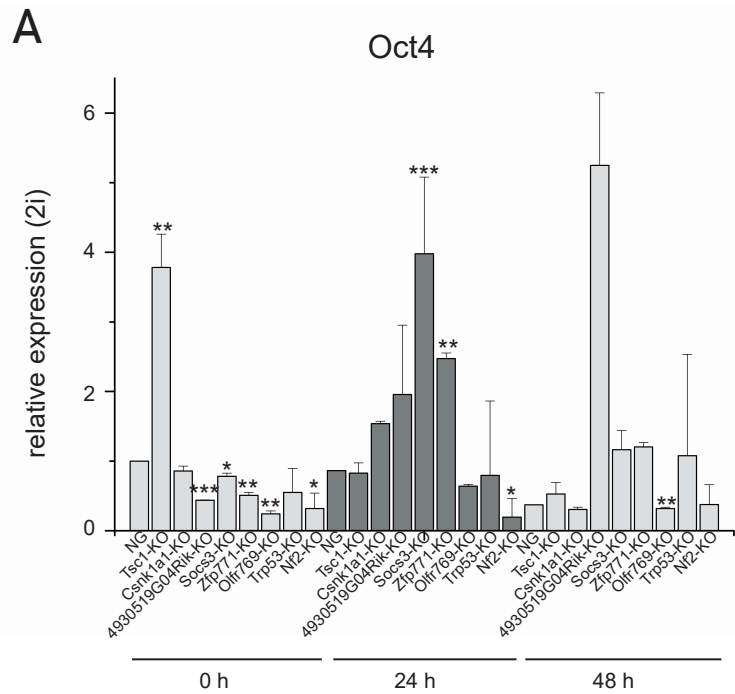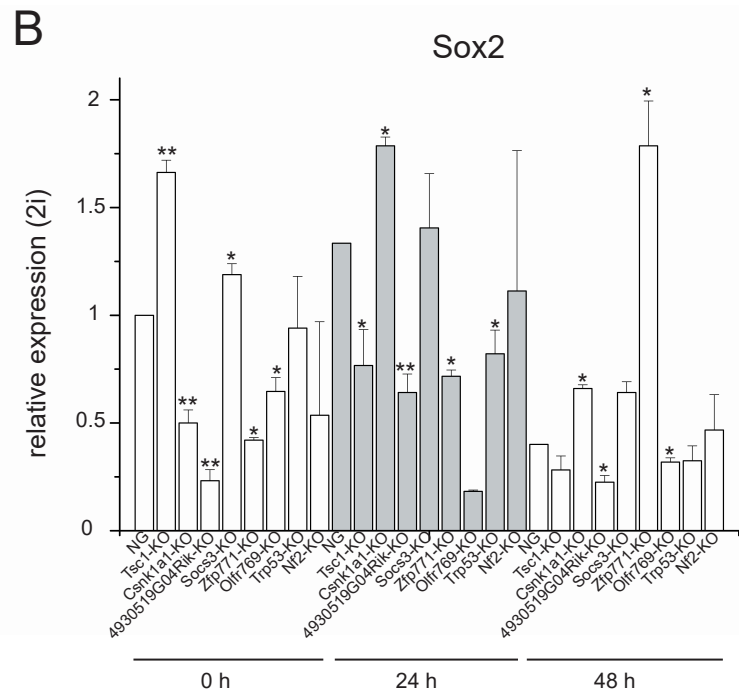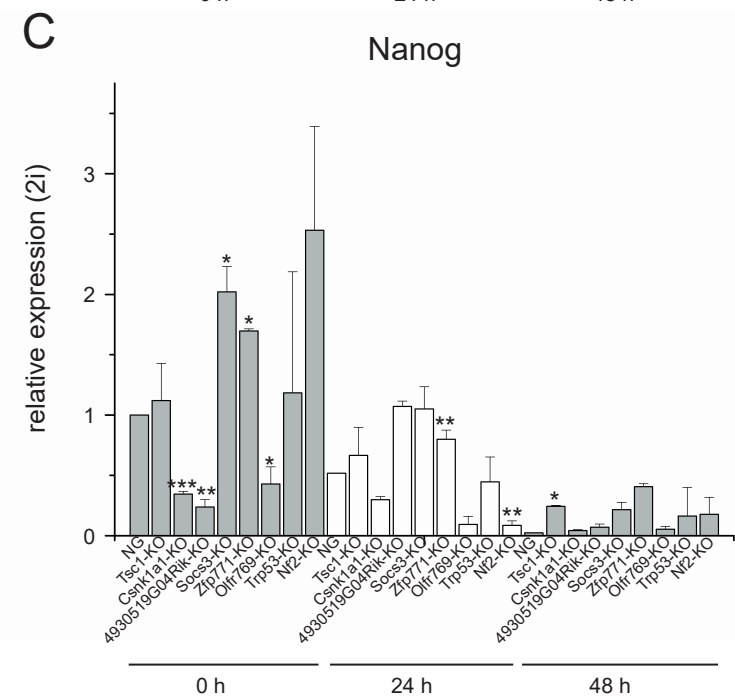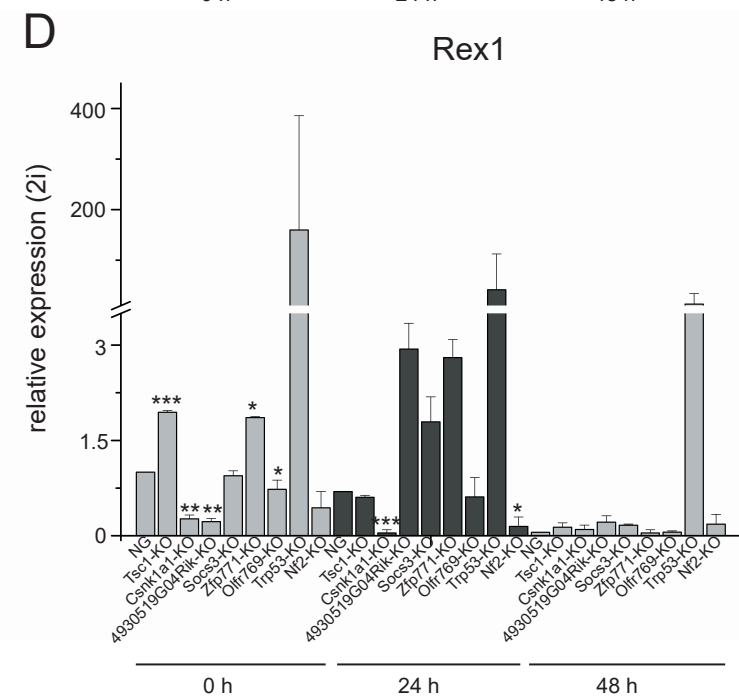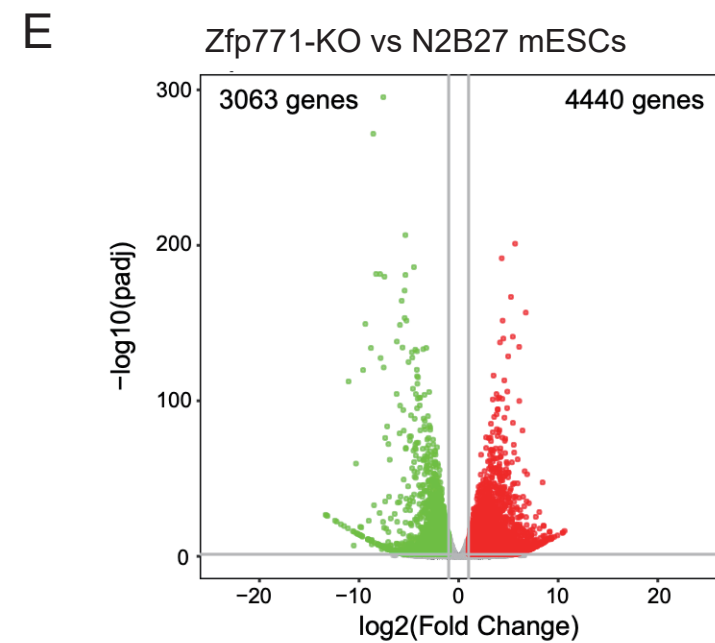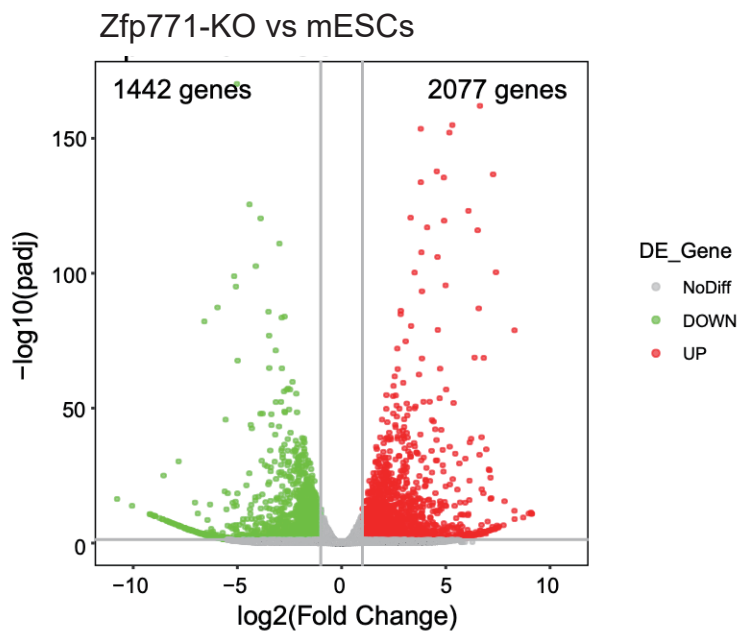

Supplement: Supplementary file 1 [file cells-11-02289-s001.zip › Figure S8.pdf]
